# Personalized identification and characterization of genome-wide gene expression differences between patient-matched intracranial and extracranial melanoma metastasis pairs

**DOI:** 10.1186/s40478-024-01764-5

**Published:** 2024-04-24

**Authors:** Theresa Kraft, Konrad Grützmann, Matthias Meinhardt, Friedegund Meier, Dana Westphal, Michael Seifert

**Affiliations:** 1 Institute for Medical Informatics and Biometry (IMB), Carl Gustav Carus Faculty of Medicine, Technische Universität Dresden, Fetscherstr. 74, 01307 Dresden, Germany; 2 Department of Pathology, Carl Gustav Carus Faculty of Medicine, Technische Universität Dresden, Fetscherstr. 74, 01307 Dresden, Germany; 3 Department of Dermatology, Faculty of Medicine and University Hospital Carl Gustav Carus, Technische Universität Dresden, Fetscherstr. 74, 01307 Dresden, Germany; 4 Skin Cancer Center at the University Cancer Center (UCC) Dresden and the National Center for Tumor Diseases Dresden (NCT), Fetscherstr. 74, 01307 Dresden, Germany; 5 National Center for Tumor Diseases Dresden (NCT), Fetscherstr. 74, 01307 Dresden, Germany

**Keywords:** Melanoma metastases, Patient-matched intra- and extracranial melanoma metastasis pairs, Personalized transcriptome analysis

## Abstract

Melanoma is the most serious type of skin cancer that frequently spreads to other organs of the human body. Especially melanoma metastases to the brain (intracranial metastases) are hard to treat and a major cause of death of melanoma patients. Little is known about molecular alterations and altered mechanisms that distinguish intra- from extracranial melanoma metastases. So far, almost all existing studies compared intracranial metastases from one set of patients to extracranial metastases of an another set of melanoma patients. This neglects the important facts that each melanoma is highly individual and that intra- and extracranial melanoma metastases from the same patient are more similar to each other than to melanoma metastases from other patients in the same organ. To overcome this, we compared the gene expression profiles of 16 intracranial metastases to their corresponding 21 patient-matched extracranial metastases in a personalized way using a three-state Hidden Markov Model (HMM) to identify altered genes for each individual metastasis pair. This enabled three major findings by considering the predicted gene expression alterations across all patients: (i) most frequently altered pathways include cytokine-receptor interaction, calcium signaling, ECM-receptor interaction, cAMP signaling, Jak-STAT and PI3K/Akt signaling, (ii) immune-relevant signaling pathway genes were downregulated in intracranial metastases, and (iii) intracranial metastases were associated with a brain-like phenotype gene expression program. Further, the integration of all differentially expressed genes across the patient-matched melanoma metastasis pairs led to a set of 103 genes that were consistently down- or up-regulated in at least 11 of the 16 of the patients. This set of genes contained many genes involved in the regulation of immune responses, cell growth, cellular signaling and transport processes. An analysis of these genes in the TCGA melanoma cohort showed that the expression behavior of 11 genes was significantly associated with survival. Moreover, a comparison of the 103 genes to three closely related melanoma metastasis studies revealed a core set of eight genes that were consistently down- or upregulated in intra- compared to extracranial metastases in at least two of the three related studies (down: *CILP*, *DPT*, *FGF7*, *LAMP3*, *MEOX2*, *TMEM119*; up: *GLDN*, *PMP2*) including *FGF7* that was also significantly associated with survival. Our findings contribute to a better characterization of genes and pathways that distinguish intra- from extracranial melanoma metastasis and provide important hints for future experimental studies to identify potential targets for new therapeutic approaches.

## Background

Patients with melanoma brain metastasis (intracranial melanoma metastasis) still have a very unfavorable prognosis. The mean overall survival of patients with untreated intracranial melanoma metastases is as little as 4 months [1], improving only to a median survival of 22.7 months with surgery followed by immunotherapy [2]. There are several emerging treatment options like targeting MAPK signaling using BRAF or MEK inhibitors or immunotherapy using anti-CTLA4/anti-PD-1 antibodies that improve patient survival. These treatment options are effective for patients with extracranial metastases [3]. For patients with intracranial metastases, the treatment response duration of BRAF/MEK inhibitors is only limited to a few months and the efficacy of immune checkpoint inhibitors is substantially reduced in symptomatic patients [1, 4–8]. Results of important clinical immunotherapy trials for melanoma patients with intracranial metastases are summarized in [9]. Clinical outcomes of melanoma patients with intracranial metastases treated with stereotactic radiosurgery and various systemic therapies have been analyzed in [10]. The overall 12 month survival rates for the combined anti-PD-1-CTLA4 therapy was 68%, 62% for BRAF/MEK inhibitor treatment, 59% for anti-PD-1 therapy, and 45% for anti-CTLA4 therapy compared to only 21% for BRAF inhibitor treatment and 15% for conventional chemotherapy. The combined treatment of patients with anti-PD-1-CTLA4 therapy showed the best median overall survival of about 21 months. The updated five year data from patients with intracranial melanoma metastases enrolled on the ABC trial investigating nivolumab plus ipilimumab or nivolumab alone confirmed the high anti-tumor activity of nivolumab plus ipilimumab in patients with asymptomatic intracranial metastases (5-year intracranial progression-free survival 52%, 5-year overall survival 55%) [11]. The NIBIT-M2 trial showed persistent therapeutic efficacy of ipilimumab plus nivolumab with a seven year overall survival rate of 42.8% in asymptomatic patients [12]. Further, in a recently published retrospective study with 376 patients with intracranial melanoma metastases treated with nivolumab plus ipilimumab, long-term survival was seen in treatment-naive, asymptomatic, steroid-free patients as well as in those patients that received stereotactic radiosurgery in combination with nivolumab plus ipilimumab [13].

Still, the therapy resistance of intracranial metastases is the leading cause of death of melanoma patients [14, 15]. Unfortunately, intracranial metastases are also very common affecting about 50% of all metastatic melanoma patients [1]. Therefore, a detailed molecular characterization of differences between intra- and extracranial metastases is needed to better characterize molecular alterations and mechanisms that distinguish both types of metastases to provide a basis for future developments of novel therapeutic strategies.

Several studies were done over the last years to identify differences between intra- and extracranial melanoma metastases. Different genetic alterations in genes like *BRAF*, *NRAS* and *CDKN2A* were suggested to contribute to a unique molecular profile of intracranial metastases [16]. Up-regulation of PI3K/Akt signaling in intracranial metastases has been reported as a key mechanism for uncontrolled proliferation of intracranial metastases [17–20]. Epigenetically regulated genes (e.g. *CSSP1*, *GRB10*, *NMB*, *PDXK*, *PRKCZ*, *RASL11B*, *STK10* and *WDR24*) with altered promoter methylation and corresponding differential expression in intra- compared to extracranial metastases were recently identified [21]. Single-cell sequencing of metastases revealed a neuronal-like cell meta-program of intracranial melanoma metastases [22]. Another single-cell sequencing study delineated brain metastases programs into a proliferative and an inflammatory archetype [23]. All these studies compared intracranial metastases from several patients to extracranial metastases from other patients. However, melanoma metastases in different organs from a patient are more similar to each other than to metastases from other patients in the same organs. This patient-specific heterogeneity of molecular data from melanoma metastases has been observed in other studies before [18, 24, 25]. Therefore, such differences between individual patients should be included in the data analysis to further improve the identification of molecular differences between intra- and extracranial melanoma metastases.

To account for the inter-patient heterogeneity, some studies already started to investigate patient-matched metastasis pairs at different molecular layers. Chen et al. [18] analyzed gene mutations, DNA copy number alterations and gene expression profiles identifying the PI3K/Akt signaling pathway as a potential therapeutic target. Fischer et al. [26] performed RNA sequencing and multiple immune-relevant sequencing analyses and identified a significant immunosuppression and enrichment of oxidative phosphorylation in intracranial melanoma metastases. We have recently performed a personalized analysis of genome-wide DNA-methylation profiles and revealed a global decrease of DNA-methylation intracranially [25]. These DNA-methylation changes between patient-matched intra- and extracranial melanoma metastases affected many genes involved in cellular signaling, growth, adhesion and apoptosis and further supported the presence of a neuronal phenotype. Further, we were also able to predict potential downstream targets of genes with altered promoter methylation, which allowed to group heterogeneous patient-matched melanoma metastasis pairs into three homogeneous subgroups utilizing a network-based approach [27]. In addition, mutations in driver genes (most frequently *ARID1A*, *ARID2* and *BRAF*), which distinguished intra- from extracranial melanoma metastases, were identified by targeted next-generation sequencing [28].

Here, we perform a personalized analysis of patient-matched gene expression profiles of intra- and extracranial melanoma metastasis pairs to account for the common developmental origin of patient-matched metastases. To realize this, each patient-matched metastasis pair was analyzed by a Hidden-Markov Model (HMM) approach to identify differentially expressed genes for each pair. This was done by transferring the recently used HMM-approach for the personalized analysis of DNA-methylation profiles by [25] to the analysis of gene expression profiles. The predicted differentially expressed genes were further used to identify frequently affected cellular pathways and to derive a set of genes that was altered in the same manner in the majority of patients. In-depth literature analysis in combination with comparisons to independent related studies were performed to provide further hints which genes potentially play an important role to establish molecular differences between intra- and extracranial melanoma metastases. Additionally, significant expression associations of several of these genes with decreased survival of melanoma patients from a large public patient cohort indicate the relevance of our findings.

## Methods

### Gene expression data of melanoma metastases

The considered gene expression data set comprises 37 melanoma metastasis samples from 16 patients. Each of these patients developed an intra- and an extracranial melanoma metastasis. The extracranial metastases included lung, lymph node, liver, skin, small intestine and soft tissue metastases. A board-certified pathologist had marked metastasis regions with a high percentage of tumor cells of at least 90% and minimal percentage of normal tissue, necrosis or hemorrhage. In addition, histologically different regions in the extracranial metastases of four patients (P04, P08, P18, P42) were included. The gene expression profiles of these distinct histological regions were included as separate patient-specific samples, because such distinct regions in a metastasis may represent different subclones within a metastasis. An overview of the data set and the patient-specific sample composition is given in Table 1. Additional information about the patients are provided in Additional file 18: Table S13. Gene expression profiles of all metastases samples were measured by RNA-sequencing (RNA-seq) and processed as described in [21]. Details to the RNA sequencing protocol and the preprocessing of the reads are provided in Additional file 22: Text S3. The considered RNA-seq data of seven patients were taken from [21] considering all patients for which transcriptomes of patient-matched intra- vs. extracranial melanoma metastasis pairs were available. The RNA-seq data of the nine other patients were additionally measured for this study to further increase the patient cohort. The expression read counts of all metastases samples were normalized with the voom function of the R-package limma performing a cyclic loess normalization [29]. To avoid the inclusion of very weakly expressed genes, only protein-coding genes of the human genome annotation (hg19) with more than one count per million (CPM > 1) in more than 25% of the metastases samples were considered. This resulted in a gene expression data set that comprised the absolute expression levels (log_2_-CPMs) of 14,946 genes in their chromosomal order across the 37 melanoma metastases samples (Additional file 6: Table S1).

### Gene expression data of normal tissues

RNA-sequencing data of 28 normal tissue samples were available to analyze the purity of the melanoma metastases samples. These normal tissue samples comprised six intracranial and 22 extracranial normal tissues (8 lymph nodes, 7 lung and 7 skin/soft tissues) from 27 patients. In total, 11 of these samples were taken from [21] and 17 samples were newly sequenced. These normal tissues were jointly normalized with the melanoma metastasis samples using the same procedure as described before. The normal tissue samples of three patients (P03, P04, P16) matched with our melanoma metastases cohort in Table 1. All considered normal tissue samples had a tumor cell content of 0%. The normalized gene expression data of the normal tissues are provided in Additional file 7: Table S2. An overview of all normal tissue samples is given in Additional file 8: Table S3.

### Hierarchical clustering of melanoma metastases and joint clustering with normal tissue

The gene expression profiles of all melanoma metastases samples (Additional file 6: Table S1) were clustered hierarchically to identify similarities and differences between the metastases. The clustering was performed using the R package pvclust [30] with 10,000 bootstrapping repetitions to obtain stability estimates for all clusters. The Manhattan distance was used as distance measure between individual samples and Ward’s minimum variance linkage method (ward.D2) [31] was used as cluster algorithm. The stability of a cluster was quantified by the approximate unbiased p-value (AU value). The AU value ranges from 0 to 100, where a larger value represents greater stability and a value of 100 means that a cluster is perfectly stable.

In addition, to further test and ensure the high tumor content in our metastasis data, all transcriptomes of metastases and normal samples were also hierarchically clustered together (Additional file 1: Figure S1). This clustering and its stability analysis were performed using the same methods as described above.

### HMM-based analysis of gene expression profiles of patient-specific metastasis pairs

Transcriptomes of metastases samples from the same patient were more similar to each other than to metastases samples of other patients. Therefore, a personalized analysis of each patient-matched intra- and extracranial melanoma metastasis pair was done to predict individual gene expression changes between the intra- and the extracranial metastasis of each pair. First, pair-specific log_2_-ratio gene expression profiles were computed for all 21 possible patient-matched pairs (Additional file 9: Table S4). Such a relative expression profile was obtained for each pair by subtracting for each gene its absolute expression (log_2_-expression value from Additional file 6: Table S1) in the extracranial metastasis from the absolute expression of this gene in the intracranial metastasis followed by sorting of the genes by their chromosomal order. The obtained log_2_-ratios quantify the expression changes of genes: (i) a log_2_-ratio clearly less than zero indicates reduced expression, (ii) a log_2_-ratio about zero indicates unchanged expression, and (iii) a log_2_-ratio clearly greater than zero indicates increased expression of a gene in the intra- compared to the extracranial metastasis of a patient-matched metastasis pair (Additional file 2: Figure S2).

Next, an autocorrelation analysis of the log_2_-ratio gene expression profiles was performed in a chromosome-specific manner. Each autocorrelation profile of each chromosome was weighted according to the number of genes that were measured on that chromosome and then averaged across all chromosomes for the corresponding lags. This weighted autocorrelation analysis revealed that genes in close chromosomal proximity tend to correlate more strongly in their expression with each other than expected by chance (Fig. 2A).

This observation motivated the usage of a Hidden Markov Model (HMM) for the personalized analysis of the expression profiles of the individual metastasis pairs, because an HMM can utilize such local chromosomal dependencies to improve the predictions of differentially expressed genes [32–35]. In more detail, the chromosome-specific log_2_-ratio gene expression profiles of all patient-specific metastasis pairs were analyzed by a standard first-order three-state HMM with state-specific Gaussian emission densities specifically developed for the analysis of individual expression profiles [33]. This HMM has three states to classify each gene according to its most likely expression state (Fig. 2B): (i) state ’−’ represents genes with decreased expression in the intracranial metastasis compared to the corresponding extracranial metastasis, (ii) state ’$=$’ represents genes with unchanged expression, and (iii) state ’$+$’ represents genes with increased expression in the intracranial metastasis compared to the corresponding extracranial metastasis of a patient-matched metastasis pair. Motivated by the distribution of the gene expression log_2_-ratios of all patient-matched metastasis pairs (Additional file 2: Figure S2), the initial means of the state-specific Gaussian emission densities were set to -3, 0, 3 and the corresponding initial standard deviations were set to 0.5, 1, 0.5 for the states ’−’, ’$=$’, and ’$+$’, respectively. In addition, the initial state probabilities were set to 0.1 for the states ’−’ and ’$+$’ and to 0.8 for state ’$=$’ to account for the fact that the vast majority of genes was unchanged in their expression. The resulting initial HMM was trained based on all patient-matched log_2_-ratio gene expression profiles using the Bayesian Baum-Welch algorithm that enables to integrate prior knowledge about expected gene expression changes into the training [32, 33]. To realize this, a grid search was done to determine hyperparameter settings for the state-specific Gaussian emission densities that led to biologically meaningful state representations of the trained HMM following the basic strategy developed in [25]. In more detail, this search was used to identify a hyperparameter setting that minimized the number of wrongly classified genes (genes with negative log_2_-ratios assigned to state ’$+$’ or genes with positive log_2_-ratios assigned to state ’−’) while maximizing the total number of genes assigned to state ’$+$’ or ’−’ to obtain a clear separation between genes with reduced and genes with increased expression. A good separation between reduced, unchanged, and increased expression levels was obtained by setting the values of the scale parameter (scaleMeans) to 2500, 1000, 2500 and those of the shape parameter (shapeSds) to 5000, 10, 5000 for the states ’−’, ’$=$’, and ’$+$’, respectively. All other hyperparameters were kept at their pre-defined standards. The training of the corresponding initial HMM took 118 iteration steps and was finished in less than one minute on a standard laptop (i7-8565U CPU: 1.80 GHz, 8 GB RAM) using the HMM implementation from [33]. State-posterior decoding was used to determine for each gene in a log_2_-ratio gene expression profile its most likely underlying expression state (Additional file 10: Table S5).

### Pathway enrichment analysis of differentially expressed genes

Enrichment analysis of differentially expressed genes was done for known cancer- and immune-relevant pathways from KEGG [36] including ’Pathways in cancer’ (hsa05200). Corresponding pathway genes were obtained using KEGGREST version 1.26.0 [37] based on the KEGG release 103.0. The obtained cancer signaling pathway annotations are listed in Additional file 11: Table S6 and the immune pathways are provided in Additional file 12: Table S7. Based on this, each gene was annotated using these pathway annotations. Next, each pathway was tested for a statistical significant enrichment of genes with decreased or increased expression in each metastasis pair using Fisher’s exact test (R function fisher.test). Each pathway was considered to be significantly enriched for decreased or increased expression when its FDR-adjusted *p*-﻿value was less then 0.05 (R function p.adjust) [38].

### Determination of differentially expressed genes shared across the majority of patients

To obtain genes that were altered in their expression in the majority of patients, a ranking of genes across all patients was done according to the number of patients in which they showed the same differential expression state. To realize this, multiple metastasis pairs from the same patient had to be summarized to count each of the four affected patients only once (Table 1). For these four patients, a majority vote for the predicted HMM expression states ’$+$’ or ’−’ was performed for each gene across the multiple metastasis pairs of the same patient. In that process, a small neglectable proportion of 0.52% of all genes had an equal number of ’$+$’ and ’−’ predictions in metastasis pairs of the patients with multiple pairs and were therefore not further considered in the analysis. Considering all patients, the genes were ranked according to their decreasing frequency of decreased expression (number of patients for which a gene was predicted to have the HMM expression state ’−’) and another ranking was made for all genes according to their decreasing frequency of increased expression (number of patients with HMM expression state ’$+$’). Additional file 13: Table S8 contains both rankings.

### Cellular functions of differentially expressed genes shared across the majority of patients

To characterize major cellular functions, a gene ontology (GO) analysis was performed considering all differentially expressed genes whose expression was altered in the same direction in at least 8 of 16 patients (Additional file 13: Table S8). This was done using clusterProfiler [39] with a targeted focus on altered biological processes. The results of this GO analysis are provided in Additional file 14: Table S9. In addition to the automatic GO enrichment analysis, a manual but in-depth hand-curated gene function and literature analysis was performed for the more stringent set of the top 103 genes that either showed increased or decreased expression in intra- compared to corresponding extracranial metastases in at least 11 of 16 patients (Additional file 13: Table S8) using UniProt [40], GeneCards [41] and Pubmed (https://pubmed.ncbi.nlm.nih.gov/).

### Independent validation of differentially expressed genes shared across the majority of patients considering three related studies

The expression behavior of the top 103 genes was compared to their expression behavior in three related studies that performed transcriptome analyses of intra- and extracranial melanoma metastases [18, 22, 26]. Therefore, the supplementary table with the corresponding top differential gene expression candidate gene set was downloaded for each of this three studies. Each of our 103 candidate genes was then checked for the presence and the direction of its differential expression in intra- compared to extracranial metastases in each of these candidate gene sets.

### Association of candidate gene expression with patient survival

Expression levels of candidate genes were tested for associations with patient survival using the melanoma cohort from The Cancer Genome Atlas (TCGA) [42] that comprises primary and metastatic melanomas. Only patient samples with available survival information and a known tumor content of at least 80% were considered. Following the TCGA gene expression data pre-processing approach in [27], the corresponding raw gene expression counts of the patients were normalized by cyclic loess normalization [29]. Next, only genes with more than one count per million (CPM) reads in at least 50% of the patients were kept to exclude lowly expressed genes. For each of the 38 candidate genes from our study that were also measured in the TCGA cohort, the TCGA patients were divided into two groups: (i) a high expression group including patients with expression of the specific candidate gene in the fourth quartile of all expression levels of this gene, and (ii) a low expression group including patients with expression levels of the specific candidate gene in the first quartile of all expression levels of the specific gene across the TCGA patients. Based on this, for each of the 38 candidate genes differences in survival between both groups were analyzed by a Kaplan-Meier analysis and tested for statistical significance by performing a log-rank test using the R package survminer version 0.4.1. The two corresponding patient groups for each gene along with the survival information are provided in Additional file 15: Table S10 and the corresponding normalized gene expression levels are contained in Additional file 19: Table S14. Each gene was considered to be significantly associated with survival if its FDR-adjusted *p*-﻿value was less then 0.1 (R function p.adjust) [38].

## Results

### Hierarchical clustering of melanoma metastasis expression profiles suggests the need for a personalized analysis of patient-matched metastasis pairs

Our cohort comprises 16 melanoma patients that all developed an intra- and an extracranial metastasis during the course of their disease (Table 1). The corresponding metastasis transcriptomes of seven patients were taken from [21] including all patients for which patient-matched transcriptomes of intra- and extracranial melanoma metastasis samples were available. In addition, the transcriptomes of metastasis pairs of nine additional patients were newly sequenced for this study. The extracranial metastases of four patients (P04, P08, P18, P42, Table 1) contained histologically distinct regions. The transcriptomes of these distinct regions from the same metastasis sample were included as separate patient-specific extracranial samples to enable the analysis of molecular differences between the potential subclones within a metastasis and in relation to their patient-matched intracranial metastasis. Further, the majority of patients (9 of 16) were not treated before metastasis resection, five of the seven pre-treated patients were neither treated with BRAF/MEK nor with immune checkpoint inhibitors, and two patients received targeted therapies (Additional file 18: Table S13). Additional file 18: Table S13 also contains the mutation status of *BRAF* and *NRAS* of the individual metastases, the sex of the patients, and the age of the patients at the time when the intracranial metastasis was resected.

First, it was reassured that the analyzed melanoma metastases had high tumor content. This was done by performing a hierarchical clustering of all initially profiled metastasis samples in a joint analysis together with transcriptomes of normal samples of different tissues comprising brain, lymph node, lung, soft tissue and skin (Additional file 1: Figure S1). The metastases and the normal tissues formed separate disjoint clusters, except for one lymph node metastasis sample of patient P06 that co-clustered together with the normal tissues. Therefore, this patient was excluded from the study.

**Fig. 1:**
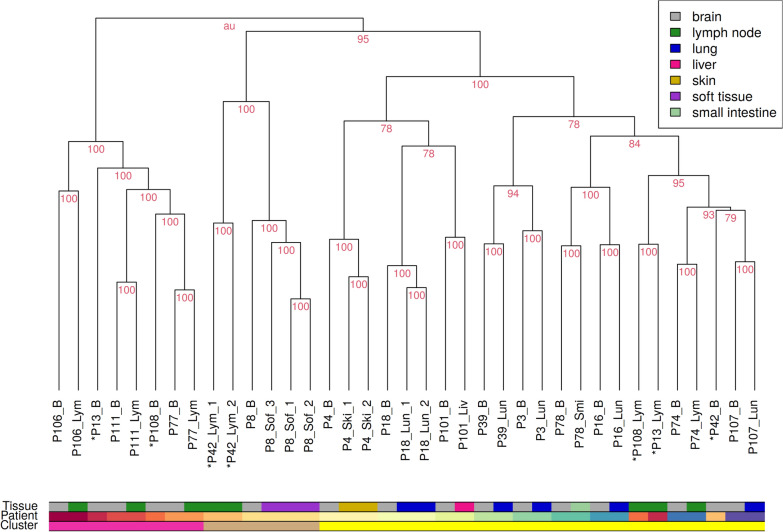
Hierarchical clustering of transcriptomes of all patient-matched melanoma metastases. Each patient developed an intracranial metastasis (B, grey) and an extracranial metastasis in the course of its disease. Extracranial metastases appeared in either lung (Lun, blue), lymph node (Lym, green), skin (Ski, yellow), liver (Liv, pink), small intestine (Smi, light green) or soft tissue (Sof, purple). Multiple samples of the same metastasis were taken if the metastasis showed histologically different regions. Metastases of the same patient mostly co-cluster together. The few exceptions from this observation are marked with an asterisk ’*’. Affiliation with one of the three main clusters is marked in the lowest color bar (left cluster: pink, middle cluster: brown, right cluster: yellow). Stability of individual clusters is quantified by the red AU value, where 100 means that a cluster was perfectly stable

Next, a hierarchical clustering of the transcriptomes of all remaining metastases was performed in combination with a cluster stability analysis to characterize global similarities and differences between all individual metastases (Fig. 1). This hierarchical clustering showed three main subclusters, which were completely stable (AU value = 100). The left subcluster (pink bar) only comprises intracranial and lymph node metastases. The middle cluster (brown bar) includes all samples from P08 and lymph node metastasis samples from P42. Further, it is important to note that P08 was the only patient that had a soft tissue metastasis. There were no associations with available patient meta-information that explained the observed co-clustering of metastasis samples of P08 and P42. The right subcluster (yellow bar) comprises metastases from six of seven extracranial tissue types (all except soft tissue).

In addition, the clustering also showed that intra- and extracranial metastases from the same patient were more similar to each other than to metastases in the same tissue from other patients. All observed patient-specific subclusters of intra- and extracranial metastasis pairs were fully stable (Fig. 1, AU value = 100). In more detail, for 13 of 16 patients, the corresponding intra- and extracranial metastases all formed completely stable patient-specific subclusters that represented patient-matched metastases co-clustered together (Fig. 1). For the other three patients, their intra- and extracranial metastases did not directly cluster together: P13 (brain and lymph node metastases), P108 (brain and lymph node metastases), and P42 (brain and lymph node metastases). Despite the fact that these three patients all developed a lymph node metastases, this observation cannot be generalized for all lymph node metastases in the cohort. The other four patients that developed a lymph node metastasis formed completely stable patient-specific subclusters that contained the corresponding intracranial metastasis co-clustered together with the patient-matched lymph node metastasis (P106, P111, P77, P74; Fig. 1).

Generally, such a patient-specific co-clustering of melanoma metastases has also been observed in closely related studies with gene expression or DNA-methylation data of patient-matched intra- and extracranial melanoma metastasis pairs [25, 26]. This observation motivates the necessity for a personalized analysis of the patient-matched metastasis pairs, which is performed subsequently.

**Table 1:** Melanoma metastasis patient and sample overview

Patient	Intracranial metastasis	Extracranial metastasis	Data origin
P03	1 brain sample	1 lung sample	Westphal et al. [21]
P04	1 brain sample	2 skin samples	Westphal et al. [21]
P08	1 brain sample	3 soft tissue samples	Westphal et al. [21]
P13	1 brain sample	1 lymph node sample	Newly sequenced
P16	1 brain sample	1 lung sample	Westphal et al. [21]
P18	1 brain sample	2 lung samples	Westphal et al. [21]
P39	1 brain sample	1 lung sample	Westphal et al. [21]
P42	1 brain sample	2 lymph node samples	Westphal et al. [21]
P74	1 brain sample	1 lymph node sample	Newly sequenced
P77	1 brain sample	1 lymph node sample	Newly sequenced
P78	1 brain sample	1 small intestine sample	Newly sequenced
P101	1 brain sample	1 liver sample	Newly sequenced
P106	1 brain sample	1 lymph node sample	Newly sequenced
P107	1 brain sample	1 lung sample	Newly sequenced
P108	1 brain sample	1 lymph node sample	Newly sequenced
P111	1 brain sample	1 lymph node sample	Newly sequenced

Each melanoma patient developed an intra- and an extracranial metastasis in the course of its disease. Extracranial metastases developed either in lung, lymph node, skin, liver, small intestine or soft tissue. Multiple samples of the same metastasis were taken if the metastasis contained histologically different regions. The corresponding metastasis transcriptomes of 7 of 16 patients were taken from our previous study by Westphal et al. [21] and the metastasis transcriptomes of 9 of 16 patients were newly sequenced. Additional information about the age of the patients at the resection of the brain metastasis, sex, therapies, and mutational states of *BRAF* and *NRAS* are provided in Additional file 18: Table S13

### Personalized analysis of the expression behavior of patient-matched metastasis pairs using a Hidden Markov Model

Due to the observed patient-specific co-clustering of metastases, the transcriptomes of the patient-matched melanoma metastases were analyzed in a personalized way to identify for each metastasis pair genes with increased or reduced expression in the intra- compared to the extracranial metastasis. To realize this, pair-specific log_2_-ratio gene expression profiles were considered (Additional file 9: Table S4). Such a pair-specific gene expression log_2_-ratio represents one of three possible expression states of a gene: (i) a value clearly smaller than zero suggests decreased expression of the specific gene in the intra- compared to the extracranial metastasis, (ii) a value of about zero indicates unchanged expression of a gene between both metastases, and (iii) a value clearly greater than zero suggests increased expression of the specific gene in the intra- compared to the extracranial metastasis.

**Fig. 2:**
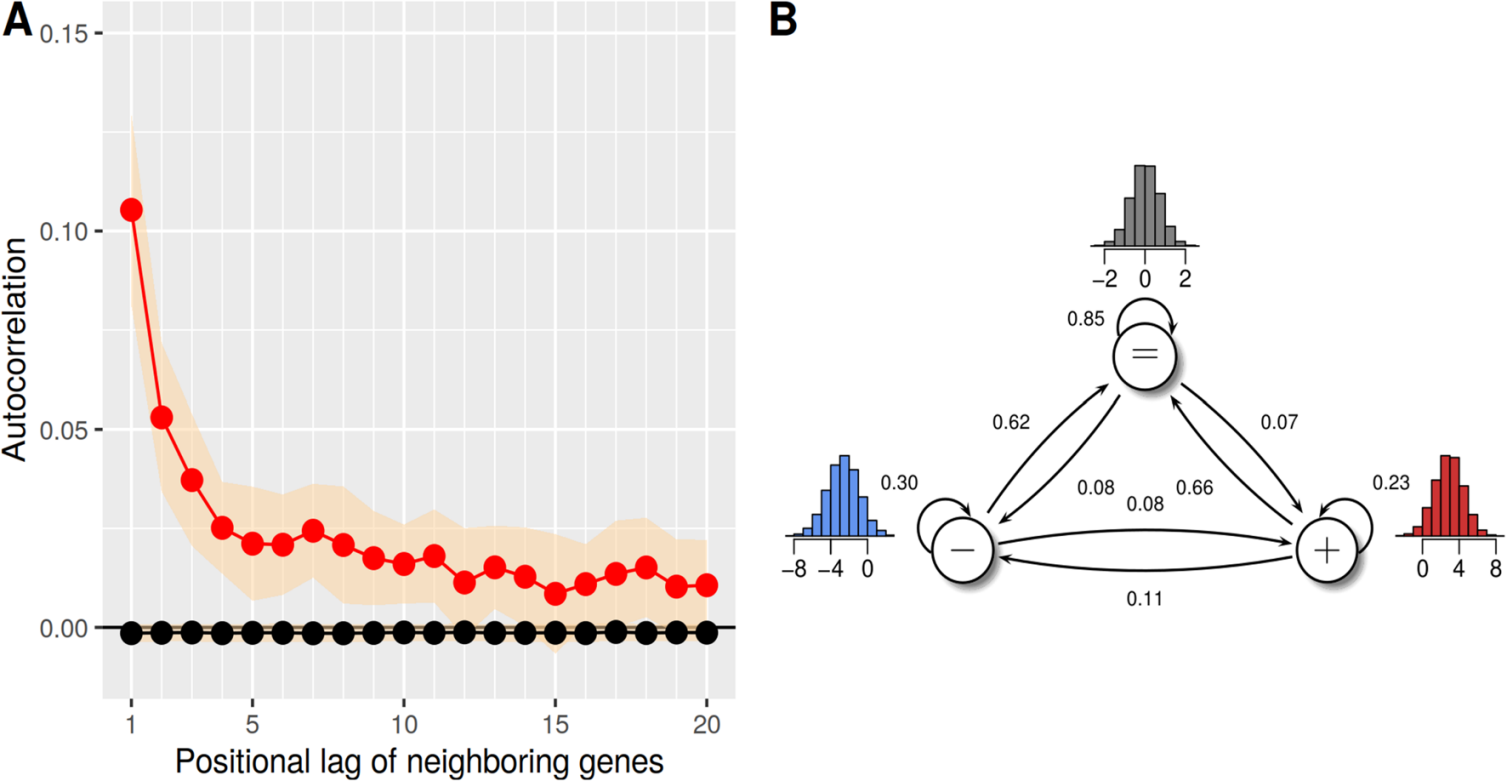
Autocorrelations of gene expression levels in close chromosomal proximity and illustration of the utilized Hidden Markov Model for decoding of gene expression states. A, Autocorrelations in the chromosomal order of genes (red) are significantly greater than the autocorrelations of 1000 randomly permuted gene expression profiles (black). The autocorrelation was calculated chromosome-wise and weighted according to the number of genes on the chromosome. Decreasing autocorrelations of log_2_-expression-ratios between intra- and extracranial metastases of genes in chromosomal order with increasing lag between the genes show that chromosomal distant genes have less similar expression than genes that are closer to each other on a chromosome. The yellow ribbon shows the standard deviations of the observed autocorrelations. B, Illustration of the three-state Hidden Markov Model (HMM) with state-specific Gaussian emission densities that was used to perform the personalized analysis of the patient-matched metastasis pairs. Genes with unchanged expression in the intra- compared to the corresponding extracranial metastasis are assigned to the state ’$=$’, genes with decreased expression in the intra- compared to the extracranial metastasis are assigned to the state ’−’, and genes with increased expression in the intra- compared to the extracranial metastasis are assigned to the state ’$+$’. The arrows that connect the states represent possible state transitions for directly neighboring genes on a chromosome and the corresponding values represent the learned state transition probabilities

The considered chromosomal log_2_-ratio gene expression profiles of the patient-matched melanoma metastases pairs show a clear positive correlation for the expression levels of genes in close chromosomal proximity (Fig. 2A). This positive autocorrelation of gene expression changes of neighboring genes motivates the usage of a Hidden Markov Model (HMM) for a personalized analysis of the patient-matched pair-specific gene expression profiles. An HMM can utilize such dependencies between neighboring gene expression levels to make reliable predictions of the underlying gene expression states. This has already been demonstrated successfully in different studies for tumor gene expression profiles (e.g. [32, 33]). For this study, a three-state HMM with state-specific Gaussian emission densities was trained to enable a personalized analysis of the individual patient-matched melanoma metastasis pairs (Fig. 2B, see Methods for details). Connected to the interpretation of the defined gene-specific log_2_-ratios above, the obtained HMM was used to assign each gene in a patient-matched metastasis pair to its most likely underlying expression state: (i) decreased expression ’−’, (ii) unchanged expression ’$=$’, and (iii) increased expression ’$+$’ in the intra- compared to extracranial metastasis.

**Fig. 3:**
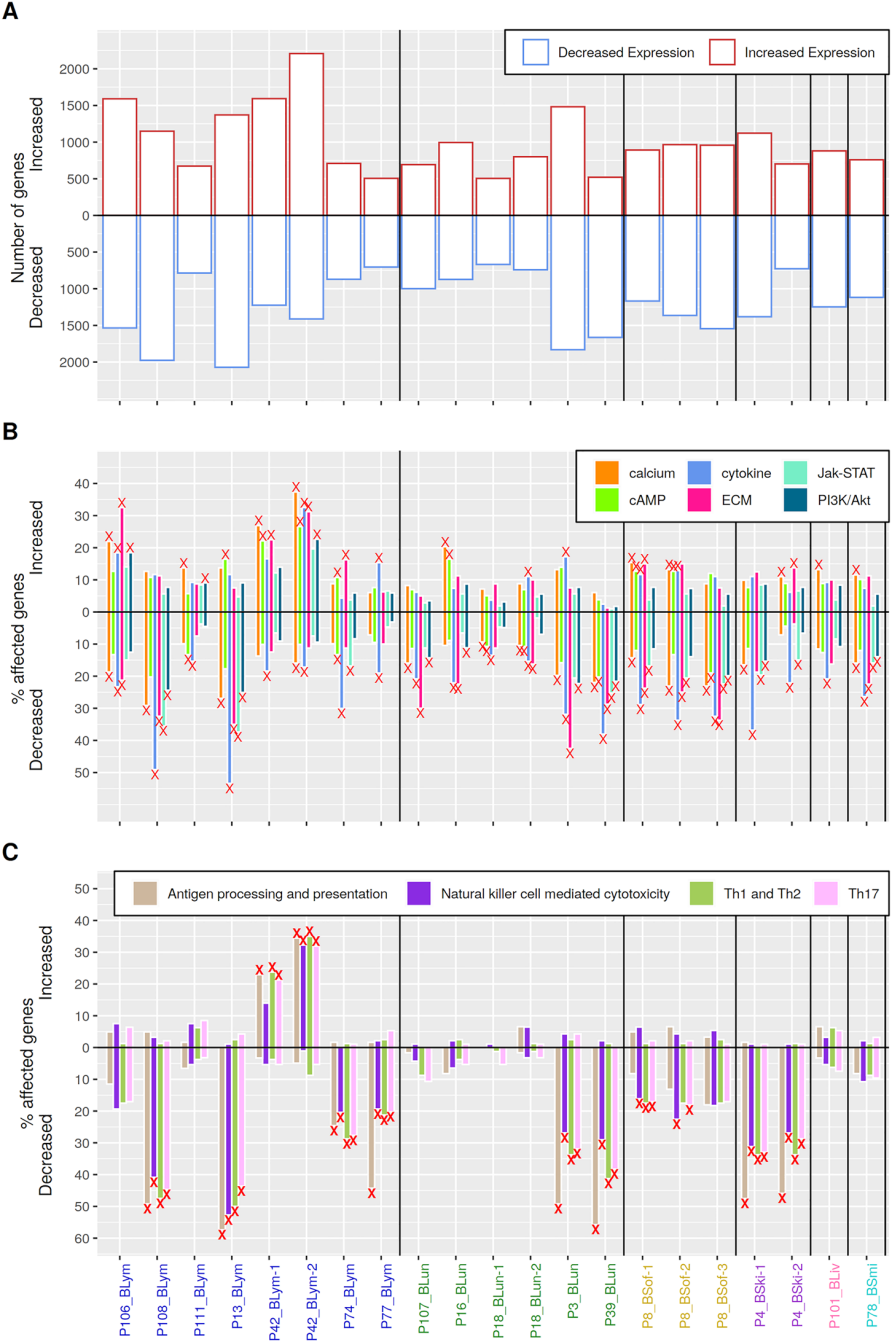
Overview of differentially expressed genes and top-ranked altered pathways across all patient-matched metastasis pairs. The x-axis shows each individual metastasis pair grouped by and color-coded according to the tissue in which the extracranial metastasis occurred (blue: lymph node, green: lung, yellow: soft tissue, purple: skin, pink: liver, cyan: small intestine). A, Absolute number of genes with increased expression in each intracranial metastasis compared to the corresponding extracranial metastasis (red bars) and the absolute number of genes with decreased expression in each intracranial metastasis compared to the extracranial metastasis (blue bars). B, Percentage of genes associated with the top six cancer-relevant signaling pathways that were either increased or decreased expressed in the intracranial metastasis compared to the corresponding extracranial metastasis of a patient-matched metastasis pair. C, Percentage of genes associated with the top four immune-relevant pathways that were either increased or decreased expressed in the intracranial metastasis compared to the corresponding extracranial metastasis of a patient-matched metastasis pair. Significant enrichment of decreased or increased expressed genes in pathways of subpanels B and C are marked with ’x’ (FDR-adjusted *p*-value﻿ < 0.05)

The numbers of predicted genes with decreased or increased expression in intra- compared to extracranial metastases are shown in Fig. 3A for each patient-matched metastasis pair. Overall, on average 1234 genes with decreased expression (standard deviation 429) and on average 1003 genes with increased expression (standard deviation 435) in intracranial metastases were found across all metastasis pairs. There was no general trend towards decreased or increased expression of genes in intra- compared to extracranial metastases across all patient-matched metastasis pairs. In more detail, the numbers of predicted differentially expressed genes varied from pair to pair (Fig. 3A: max/min of 2192/504 genes with increased expression for P42_BLym-2/P77_BLym; max/min of 2047/693 genes with decreased expression for P13_BLym/P77_BLym).

### Characteristic expression alterations of cancer-relevant signaling pathways of individual patient-matched metastasis pairs

The HMM was used to predict differentially expressed genes for each patient-matched melanoma metastasis pair (Fig. 3A, Additional file 10: Table S5). These predictions formed the basis for an individual metastasis pair-specific gene enrichment analysis to analyze which known cancer-relevant signaling pathways were significantly affected by gene expression alterations in each metastasis pair (Additional file 3: Figure S3A). Every metastasis pair was enriched for differential expression of at least one cancer-relevant signaling pathway. Overall, there were six known cancer-relevant signaling pathways (cytokine-receptor interaction, calcium signaling, ECM-receptor interaction, cAMP signaling, Jak-STAT and PI3K/Akt signaling) that were frequently affected by differential expression in at least ten metastasis pairs (Fig. 3B). More details to specific pathway overrepresentations of individual metastasis pairs are provided in Additional file 20: Text S1.

### Characteristic expression alterations of immune-relevant pathways in individual patient-matched metastasis pairs

Other interesting pathways for an analysis of differentially expressed genes between patient-matched intra- and extracranial melanoma metastases are immune signaling pathways. Therefore, a similar enrichment analysis was done for the pair-specific differentially expressed genes predicted by the HMM with a focus on known cancer-relevant immune signaling pathways (Additional file 3: Figure S3B, Fig. 3C). Significant enrichments of differential expression in immune pathways were almost always only observed for genes with decreased expression in the intra- compared to the corresponding extracranial metastasis of the patient-matched metastasis pairs, except for patient P42, who showed an enrichment of immune signaling genes with increased expression in the intracranial metastasis. These enrichments of genes with decreased expression were not restricted to a specific tissue type in which the extracranial metastases occurred. Further, no significant enrichments of immune signaling pathways were found for eight metastasis pairs of seven patients (P106_BLym, P111_BLym, P107_BLun, P16_BLun, P18_BLun-1, P18_BLun-2, P111_BLiv, P78_BSmi). Generally, four pathways were most frequently significantly enriched for differentially expressed genes across all patient-matched metastasis pairs (Fig. 3C: Antigen processing and presentation, Natural killer cell mediated cytotoxicity, Th1 and Th2, Th17 pathway). All these pathways showed an enrichment of genes with decreased expression in the intracranial metastases for the majority of patients, except for patient P42 which showed an enrichment of genes with increased expression. Details to specific immune pathway overrepresentations of individual metastasis pairs are summarized in Additional file 21: Text S2. Patient P42, the only patient with a significant overexpression of immune signaling, had a *NRAS* mutation present in both metastases. Roughly a quarter of the patients that did not show significant enrichments of immune signaling had a *BRAF* mutation. A bit more than a third of the patients that showed significant downregulations of immune signaling had a *BRAF* and/or *NRAS* mutation (Additional file 18: Table S13).

### Multiple patient-matched metastasis pairs of histologically different regions are highly similar to each other

**Fig. 4:**
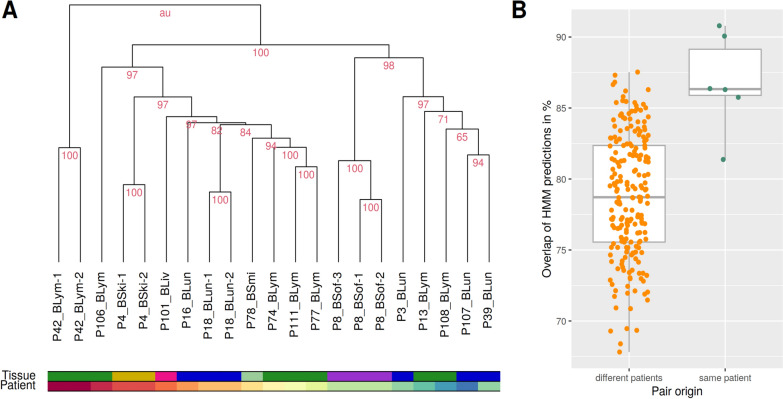
Similarities of gene expression alterations of metastasis pairs from the same and different patients. A, Hierarchical cluster dendrogram representing the similarities of genome-wide log_2_-ratio gene expression profiles comparing the intra- to the corresponding patient-matched extracranial metastasis sample. The color bars represent the tissue type in which the extracranial metastases occurred (upper bar, blue: lung; green: lymph node, yellow: skin, purple: soft tissue, pink: liver, light green: small intestine) and highlight the patient number by a color code (lower bar). Multiple metastasis pairs from the same patient are labeled with a number at the end of the corresponding identifier of the metastasis pair (see Table 1). Multiple metastasis pairs of each patient formed distinct clusters. Stability of individual clusters is quantified by the red AU value, where 100 means that a cluster was perfectly stable. B, Overlaps of the HMM-based predictions of differentially expressed genes for all pairwise comparisons of either two metastasis pairs from different patients (left boxplot) or for two metastasis pairs from the same patient (right boxplot). Overlap of HMM predictions are significantly different between pairs from different and same patients (Mann–Whitney-U-test: *p* < 0.003)

The analyzed melanoma metastases cohort contains four patients with multiple melanoma metastasis pairs that assign the intracranial metastasis of a specific patient to histologically different regions in the corresponding extracranial metastasis of this patient (Table 1: P04, P08, P18, P42). These histologically different regions within the extracranial metastases were marked by an experienced board-certified pathologist to enable separate analyses. In Fig. 3, it is clearly noticeable that the metastasis pairs from the same patient show very similar alteration patterns for enrichment of differentially expressed genes in signaling and immune pathways. Therefore, similarities of genome-wide gene expression alterations from patients with multiple metastasis pairs were analyzed in more detail. Hierarchical clustering of the patient-matched pair-specific gene expression log_2_-ratio profiles comparing the intra- to the corresponding extracranial metastasis showed that metastasis pairs of patients with multiple marked extracranial regions from histologically different regions always co-clustered together in a patient-specific manner (Fig. 4A). Further, the multiple metastasis pairs from the same patients showed a significantly greater overlap of predicted gene expression states than non-patient-matched intra- versus extracranial metastasis pairs (Fig. 4B, two-sided Mann–Whitney-U-test: *p* < 0.003, median 86.3% vs. 78.7% overlap, Additional file 16: Table S11). Thus, despite histologically different regions in some extracranial metastases, the corresponding metastasis pairs of patients with multiple extracranial samples still showed highly patient-specific expression profiles. These expression profiles are more similar to each other than to expression profiles of patient-matched pairs or non-patient-matched pairs from other patients.

### Gene ontology analysis identifies biological processes frequently affected by decreased and increased expression of genes in patient-matched metastasis pairs

**Fig. 5:**
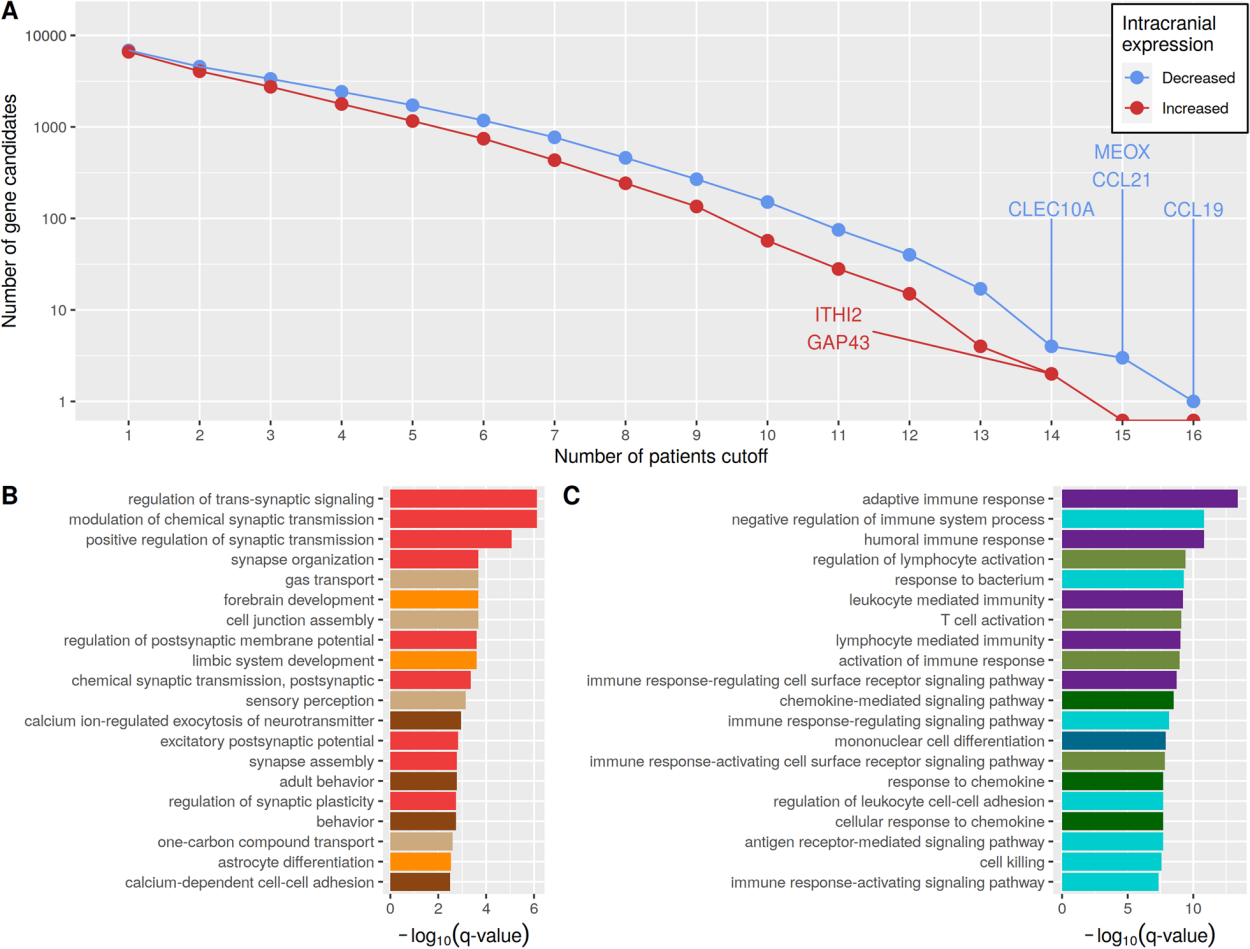
Overlap of differentially expressed genes between melanoma patients and gene ontology (GO) enrichment analysis of top differentially expressed genes. A, Number of candidate genes that were shared across multiple patients. The x-axis shows the number of patients that commonly share the corresponding differentially expressed genes. The y-axis shows the total number of shared differentially expressed genes (shared genes with decreased (blue)/increased (red) expression in patient-matched intra- compared to extracranial metastasis pairs). B, GO analysis of biological processes of the 242 candidate genes with increased expression in intracranial metastases compared to their corresponding extracranial metastases that were shared across at least eight or more patients. Colors of the bars mark biological process groups (red: synaptic processes, orange: brain-specific cell development, dark brown: neuronal processes, light brown: no specific group). C, GO analysis of biological processes of the 459 candidate genes with decreased expression in intracranial metastases compared to their corresponding extracranial metastases that were shared across at least eight or more patients. Colors of the bars mark biological process groups (purple: immune response, green: activation of immune system, darkgreen: chemokine-related processes, blue: immune-cell development, light blue: other immune-related processes)

Next, genes that shared the same differential expression across multiple patients were determined. Since metastasis pairs of the same patient showed very similar expression behavior (Figs. 3 and 4), HMM-based gene expression state predictions from the metastasis pairs of the same patient were summarized to one expression state per gene (see Methods). This allowed to include each patient only once in the ranking of genes to avoid patient-specific biases. The genes were separately ranked according to their number of increased expression state predictions (state ’$+$’ predicted by HMM) across the intracranial metastases of the patients and according to their number of decreased expression state predictions (state ’−’ predicted by HMM). Both rankings are provided in Additional file 13: Table S8. In general, more genes with decreased expression than genes with increased expression were observed for all patient cutoffs (Fig. 5A). Only one gene, *CCL19*, showed decreased expression in intra- compared to extracranial metastases in all 16 patients. *CCL19* is a cytokine involved in immunoregulatory and inflammatory processes indicating that these processes are potentially downregulated in intracranial metastases. None of the genes shared increased expression in the intra- compared to the corresponding extracranial metastasis in 15 or more patients, but there were two genes, *ITIH2* and *GAP43*, which showed increased expression in 14 patients. *ITIH2* contributes to stability of the extracellular matrix and *GAP43* is a growth factor highly expressed in neuronal growth.

To further analyze biological processes of the top differentially expressed genes that shared the same expression state in at least 50% (8 of 16) or more of all patients, the corresponding 242 genes with increased expression and the corresponding 459 genes with decreased expression genes were considered for a gene ontology enrichment analysis [39] (Additional file 14: Table S9).

Interestingly, the 242 genes that showed increased expression in patient-matched intra- compared to extracranial melanoma metastasis were frequently involved in synaptic processes (e.g. regulation of trans-synaptic signaling, synapse organization), brain-specific cell development (e.g. forebrain development, gliogenesis, astrocyte differentiation) and neuronal processes (e.g. sensory perception, calcium ion-regulated exocytosis of neurotransmitter, adult behavior) (Fig. 5B). The observation of such a brain-like phenotype for intracranial melanoma metastases is supported by other related studies including single-cell RNA sequencing [22, 43] and was also found in our corresponding previous genome-wide DNA-methylation analysis of the patient-matched metastasis pairs [25]. An additional analysis of the gene expression levels of the genes associated with the brain-like phenotype by directly comparing our intracranial melanoma metastases to our normal brain tissues showed significantly lower expression for 63.5% and significantly increased expression for 6% of the genes in the intracranial metastases, whereas 30.5% of the genes were expressed at the same level like in normal brain tissues (Additional file 4: Figure S4). This indicates that the observed brain-like phenotype is potentially jointly driven by normal cells in the metastases microenvironment and tumor cells of the intracranial metastases.

Further, the 459 genes that showed decreased expression in patient-matched intra- compared to extracranial metastasis were frequently involved in immune responses (e.g. humoral immune response, adaptive immune response, positive regulation of immune response), immune-cell development (e.g. leukocyte differentiation) and chemokine-related processes (e.g. chemokine-mediated signaling, response to chemokine) (Fig. 5C). This is also supported by a related study [26], which identified significant immunosuppression in intracranial melanoma metastases.

Overall, this suggests a down-regulation of immune-related processes and an up-regulation of processes involved in the establishment of a brain-like phenotype in patient-matched intra- compared to extracranial metastasis pairs.

### In-depth analysis of most frequently differentially expressed genes in patient-matched intra- compared to extracranial metastasis pairs

**Fig. 6:**
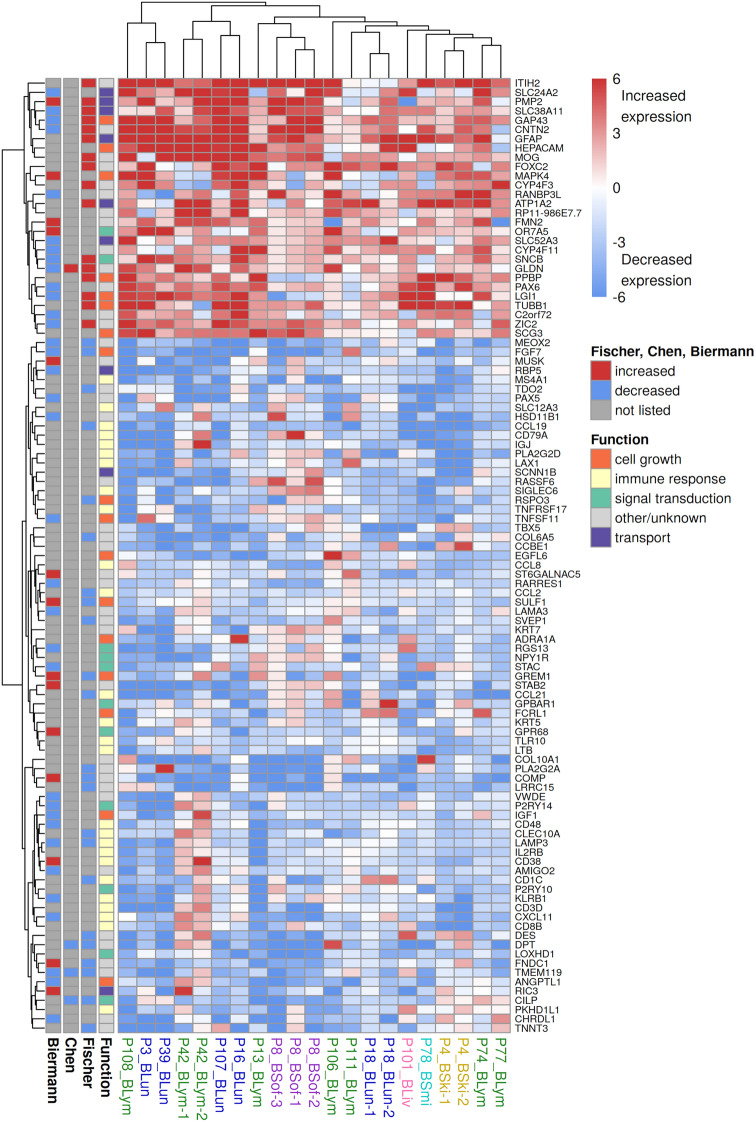
Heatmap of gene expression differences of most frequently altered genes between patient-matched intra- and extracranial melanoma metastasis pairs. The heatmap shows the expression behavior of the 28 genes with increased and the 75 genes with decreased expression state predictions by the HMM that were altered in at least 11 or more patients. A color gradient from blue over white to red is used to visualize the log_2_-ratios of the gene expression levels of the intra- compared to the corresponding extracranial metastasis of each patient-matched metastasis pair. The columns of the heatmap represent the individual metastasis pairs with labels color-coded by the tissue in which their extracranial metastasis occurred (blue: lung, green: lymph node, purple: soft tissue, yellow: skin, pink: liver, cyan: small intestine). The rows of the heatmap represent the individual candidate genes. The annotation block on the left side of the heatmap shows general gene function groups of individual genes and the expression behavior of each gene in three related studies (Fischer et al. [26], Chen et al. [18] and Biermann et al. [22])

The identification of genes that are altered in the same manner in their expression between intra- and extracranial melanoma metastases is an important step to identify potential key genes that contribute to the establishment of molecular differences between both metastasis types. Therefore, a more stringent top candidate gene set was created that consisted of genes that were differentially expressed in intra- compared to extracranial metastasis in 11 or more patients. This resulted in 103 genes comprising 75 genes with decreased and 28 genes with increased expression in intracranial metastases. Most of these 103 candidate genes showed a homogeneous expression behavior across all or the majority of metastasis pairs and were further manually assigned to more specific cancer-relevant functional categories by an in-depth gene function and literature analysis (Fig. 6). The expression levels of the candidate genes in intracranial metastases were further compared to normal brain tissues to provide hints how tumor cells of the intracranial metastases and normal cells of the microenvironment may express individual genes (Additional file 5: Figure S5).

Considering the 75 genes with decreased expression in intracranial metastasis in relation to their functional annotations (Fig. 6, lower blue block), several of these genes (26/75) are involved in immune response (e.g. *CCL19*, *CLEC10A*, *CD8B*, *CD79A*). Ten genes are involved in cell growth (*FGF7*, *RSPO3*, *TNFSF11*, *EGFL6*, *SULF1*, *ADRA1A*, *GREM1*, *FCRL1*, *IGF1*, *ANGPTL1*), and another nine genes are involved in signal transduction (*GPR68*, *NPY1R*, *LOXHD1*, *P2RY10*, *RGS13*, *CILP*, *STAC*, *P2RY14*, *GPBAR1*).

Considering the 28 genes with increased expression in intracranial metastasis in relation to their functional annotations (Fig. 6, upper red block), seven of them are involved in cell growth (*GAP43*, *HEPACAM*, *MAPK4*, *PPBP*, *LGI1*, *TUBB1*, *SCG3*). Six genes are involved in cellular transport processes (*PMP2*, *SLC38A11*, *SLC52A3*) frequently including ion transport (*GFAP*, *SLC24A*, *ATP1A2*). Three genes are involved in cell adhesion (*MOG*, *CNTN2*, *GLDN*), and three other genes (11%) are involved in metabolism (*ITIH2*, *CYP4F11*, *CYP4F3*).

### Comparison of the most frequently differentially expressed genes to three related studies

In addition, we compared our candidate gene set of 103 genes to the candidate gene sets of three closely related melanoma metastasis expression studies that compared intra- to extracranial metastases (see Methods for details, Fig. 6, Additional file 17: Table S12). There was a significant overlap of our candidate gene set with the candidate gene sets of the three related studies (Fischer et al. [26]: 38% overlap, *p* < 0.001; Chen et al. [18]: 3.8% overlap, *p* $=$ 0.001; Biermann et al. [22]: 47% overlap, *p* < 0.001; Fisher’s exact test). Further, the direction of expression changes between intra- and extracranial metastases were the same for all overlapping genes with the related bulk gene expression studies from Fischer et al. [26] and Chen et al. [18], but differed for some genes for the single-cell gene expression study by Biermann et al. [22].

The genes that were consistently found in multiple independent studies are potentially the most relevant candidate genes for future experimental studies. Therefore, the eight genes (*MEOX2*, *FGF7*, *DPT*, *LAMP3*, *CILP*, *TMEM119*, *PMP2*, *GLDN*) that were predicted as differentially expressed between intra- and extracranial metastases in our and at least two of the three related studies (Fig. 6) were summarized in Table 2 to provide an overview of their expression behavior, biological functions, and associations with cancer.

*TMEM119* was found to be down-regulated in intracranial metastases in all three related studies. *TMEM119* is involved in immune response and frequently used as a microglia marker [44]. Other genes with decreased expression in intracranial metastases include *MEOX2* involved in transcription, *FGF7* involved in cell growth, *DPT* involved in adhesion, *LAMP3* involved in immune response and *CLIP* involved in signal transduction. Further, two genes, *PMP2* and *GLDN*, were found to be increased expressed in intracranial metastases in our and two other related studies. *PMP2* is involved in transport processes of the peripheral nervous system and *GLDN* is involved in cell adhesion. Moreover, all eight genes have known associations to different kinds of cancer (Table 2). The genes are associated with laryngeal cancer (*MEOX2* [45]), cervical cancer (*FGF7* [46]), oral cancer (*DPT* [47]), breast cancer (*CILP* [48]) or ovarian cancer (*TMEM119* [49]) and *LAMP3*-positive dendritic cells are generally associated with cancer [50]. Further, both genes with increased expression in intracranial metastases (*PMP2* and *GLDN*) have known associations with melanoma cell invasion and mutation [51, 52].

**Table 2:** Summary of in-depth literature analysis of gene candidates that were also found to be differentially expressed in at least two of the closely related melanoma studies

Gene	’$+$’	’−’	Support	Biological function	Cancer association	References
MEOX2	0	15	F, B	transcription	Promotes apoptosis by PI3K/Akt pathway in laryngeal cancers	[45]
FGF7	1	13	F, B	cell growth	Inhibition leads to inhibition of cell proliferation in cervical cancer	[46]
DPT	2	13	F, C	cell adhesion	Metastasis predictor of oral and endometrial cancer, regul. tumor invasion and metastasis	[47, 53]
LAMP3	0	11	F, B	immune response	LAMP3^+^ dendritic cells associated with cancer	[50]
CILP	1	11	F, C	signal transduction	Immune infiltration in breast cancer metastasis	[48]
TMEM119	0	11	F, B, C	immune response	Facilitates ovarian cancer cell proliferation, promotes gastric cancer cell migration	[49, 54]
PMP2	11	2	F, B	transport	Drives melanoma cell invasion	[51]
GLDN	11	0	F, C	cell adhesion	Mutation in melanoma	[52]

The first column shows the gene name. The second and the third column show the number of patients where a specific gene was predicted by the HMM to have increased ’$+$’ or decreased ’−’ in expression in the intra- compared to the extracranial metastasis of patient-matched metastasis pairs. The fourth column lists the related studies (F: Fischer et al. [26], B: Biermann et al. [22] and C: Chen et al. [18]) where the gene was also reported as a gene candidate that distinguishes intra- from extracranial metastases. The expression changes of the genes in the intracranial metastases of the three related studies were the same as those found for the majority of our patients, except for *PMP2* and *GLDN* for which the expression behavior in the single-cell study from Biermann et al. [22] differed. The fifth column summarizes the biological function of the genes. The sixth and seventh column show known connections to cancer and melanoma along with corresponding references

### Expression of several candidate genes is significantly associated with survival of melanoma patients

Finally, the analysis of the 103 candidate genes that differed in their expression between the intra- and corresponding extracranial metastasis for at least 11 of 16 patients was extended to the publicly available melanoma cohort from The Cancer Genome Atlas (TCGA) [42] to analyze if the expression behavior of at least some of these genes is associated with patient survival. This can reveal genes that might also contribute to poor survival of melanoma patients with intracranial metastases.

**Fig. 7:**
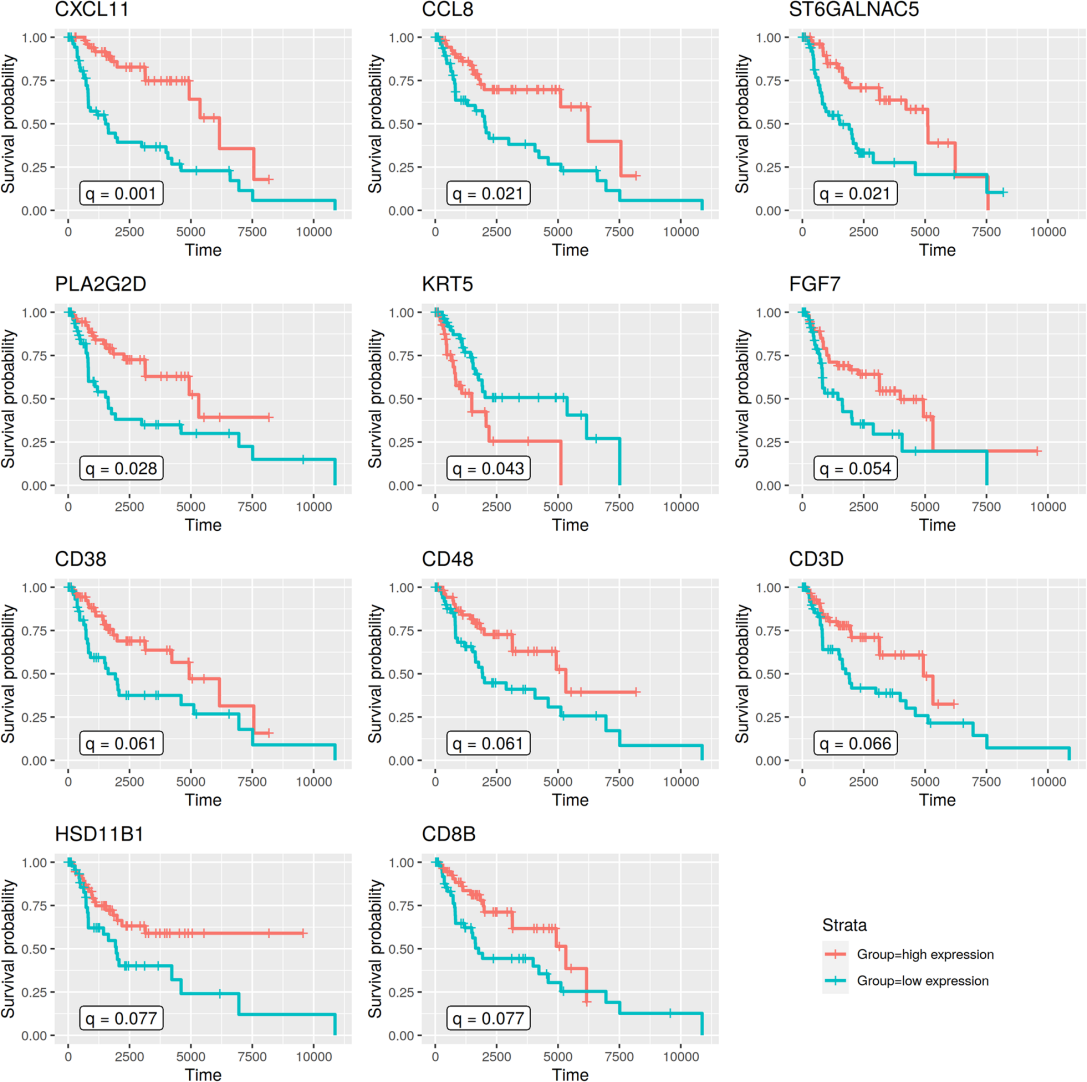
Survival analysis of melanoma patients from TCGA based on candidate genes that differed in their expression between intra- and extracranial metastasis pairs. Kaplan-Meier curves show differences in survival between melanoma patients from TCGA in the first quartile (blue line, low expression group) and the fourth quartile (red line, high expression group) based on the expression of the specific candidate gene across all TCGA melanoma patients. Differences in survival were statistically tested using the log-rank test and adjusted for multiple testing across all 38 gene candidates by computing FDR-adjusted *p*-values (q-values). Only significant results are shown (FDR-adjusted *p*-value: q < 0.1)

Only patient samples with a reported tumor content of at least 80% and available survival information were considered from the TCGA melanoma cohort. In total, 228 melanoma patients fulfilled this comprising their 34 primary melanoma samples and 194 metastatic melanoma samples. Among the 194 metastatic melanoma samples were 120 regional lymph node metastases, 42 regional skin or soft tissue metastases, 30 distant metastases, and two metastases without additional information. Each sample included expression values of 38 of our 103 candidate genes. These genes were analyzed for an association between gene expression and patient survival using a Kaplan-Meier analysis in combination with a log-rank test. For this analysis, the considered patients from TCGA were divided into two distinct groups for each of the 38 genes based on the expression of each individual gene across all TCGA patients: (i) a high expression group for patients with expression levels of the corresponding gene in the fourth quartile (Fig. 7, red curves), and (ii) a low expression group for patients with corresponding expression levels of the gene in the first quartile (Fig. 7, blue curves).

Overall, 11 of 38 candidate genes that were present in the melanoma cohort from TCGA were significantly associated with survival (Fig. 7, FDR-adjusted *p*-﻿value < 0.1). Most of these genes (8/11, 73%) are involved in immune response (*CD8B*, *CD38*, *PLA2G2D*, *KRT5*, *CCL8*, *CD48*, *CD3D*, *CXCL11*). Another two of them (2/11, 18%) are involved in metabolism (*HSD11B1*, *ST6GALNAC5*), and *FGF7* is involved in cell growth. All of these genes showed decreased expression in intra- compared to extracranial metastasis pairs. Ten of 11 genes also showed shorter survival for decreased gene expression (Fig. 7). *KRT5* was the only gene that was associated with longer survival for decreased expression.

Three of these genes are reported to play a role in melanoma. *CCL8* is involved in melanoma metastasis formation [55] and the expression of this gene was found to be correlated with immune infiltration [56]. *CD38* regulates outgrowth of primary melanoma [57]. A knockdown of *KRT5* significantly increased melanoma cell migration and invasion [58].

In summary, 11 of our 38 candidate genes that were present in the TCGA melanoma cohort were significantly associated with patient survival. This suggests that a reduced expression of these genes in intracranial metastases may potentially also contribute to the poor prognosis of intracranial metastases.

## Discussion

Melanoma brain metastases still present a major clinical challenge compared to extracranial metastases [59]. Therefore, the identification of driver genes and pathways that distinguish intra- from extracranial metastases is an important next step to provide a basis for the development of new therapeutic strategies. To contribute to this, we analyzed gene expression profiles of melanoma metastases from 16 patients by utilizing a computational strategy for the personalized analysis of individual patient-matched melanoma metastasis pairs.

In a first analysis step, we could show that melanoma metastases are clearly more similar to other melanoma metastases of the same patient than to melanoma metastases from different patients in the same tissue. The corresponding patient-specific clustering of gene expression profiles of melanoma metastases can be accounted to the common evolutionary development of patient-matched metastases from the same primary tumor. A very similar behavior of melanoma metastases has previously been observed for gene expression data [26] and DNA-methylation profiles of melanoma metastases [25]. Such a patient-specific clustering of melanoma metastases samples suggested the strong need for a personalized analysis of patient-specific melanoma metastasis pairs. This was realized by using a three-state Hidden Markov Model (HMM) approach to predict the most likely underlying gene expression state of each gene in each metastasis pair. HMMs are valuable tools to classify molecular alterations that distinguish intra- from extracranial melanoma metastases in individual patients. This has recently been demonstrated for the prediction of DNA-methylation changes of patient-matched melanoma metastasis pairs [25] and was also previously shown to work well for the identification of differentially expressed genes in individual tumor expression profiles [32, 33].

The individual prediction of differentially expressed genes for each patient-matched melanoma metastasis pair provided by the HMM was used to analyze individual enrichments of differential expression in cancer-related pathways. This revealed six cancer-associated signaling pathways that were frequently enriched for differential expression (cytokine-receptor interaction, calcium signaling, ECM-receptor interaction, cAMP signaling, Jak-STAT, and PI3K/Akt signaling).

PI3K/Akt signaling has already been frequently reported to play an important role in melanoma brain metastasis [17–19, 25, 60]. Further, cytokine-receptor interaction pathway genes and ECM pathway genes were reported to be differentially methylated in melanoma brain metastasis [25] and cytokine receptors have been reported to be significantly differentially expressed in melanoma cell lines [61]. Further, only little is known about the role of the cAMP signaling pathway in regard to melanoma. However, there is some evidence that cAMP signaling plays an important role in melanoma through a link to the MAPK pathway [62]. Moreover, the calcium signaling pathway was also frequently significantly enriched for differentially expressed genes and has been reported to influence tumor cell proliferation, invasion and cell death [63]. Recently, calcium signaling was reported to trigger communication between glioblastoma tumor cell networks with impacts on tumor cell viability and tumor growth [64]. In our cohort, a significant enrichment of the calcium signaling pathway genes with increased expression in intra- compared to extracranial metastases was found for every patient-matched metastasis pair. In addition, our candidate genes with increased expression in patient-matched intra- compared to extracranial metastasis pairs, which were shared across 50% of all patients, were enriched for functional terms associated with a brain-like phenotype. These results are also supported by the identification of a brain-like phenotype in other related studies [22, 25, 43]. Our additional direct comparisons of intracranial metastases to normal brain tissues further suggest that the observed brain-like phenotype is potentially jointly driven by normal cells in the metastases microenvironment and tumor cells of the intracranial metastases. Especially the genes predicted to be significantly up-regulated in the intracranial metastases compared to the normal brain tissues could be interesting candidates for further experimental validations of the brain-like phenotype (*CDH15*, *GJC3*, *MSX1*, *OR7A5*, *ZIC1*).

Immune-relevant pathways were almost exclusively (except patient P42) enriched for decreased expression in intracranial metastases in the personalized analysis of the patient-matched metastasis pairs by the HMM. Importantly, this downregulation of immune pathway genes in intracranial metastases was found in relation to all types of extracranial metastasis independent of the fact in which tissue they occurred. This observation was also supported by the gene ontology analysis of the top-ranked decreased genes shared across multiple patients, which included many immune-related terms. Such an underexpression of immune-relevant genes in bulk samples of intracranial metastases could be associated with the blood brain barrier, but it has previously been reported that this barrier is compromised in melanoma patients with intracranial metastases [65, 66]. Further, in accordance with our study, a significant immunosuppression in intracranial melanoma metastases was previously reported in a closely related study [26]. Thus, a downregulation of immune pathways may contribute to the poor prognosis of intracranial metastasis.

Next, we derived a candidate set of genes that consistently showed increased or decreased expression in intracranial metastases of multiple patients. This candidate gene set was compared to the candidate gene sets of three related melanoma metastases studies [18, 22, 26] to determine how frequently individual altered genes were observed in other transcriptome analyses. Overall, a total of eight of our candidate genes (down: *CILP*, *DPT*, *FGF7*, *LAMP3*, *MEOX2*, *TMEM119*; up: *GLDN*, *PMP2*) were also differentially expressed in the same direction in two or three of the related studies. All of these genes were reported to play a role in cancer (Table 2). These genes could therefore be of great interest for future experimental studies. This gene set contained *TMEM119*, which was the only gene that was altered in the same way in all three related studies and our study. *TMEM119* can be used as a marker for microglia [44]. However, microglia are predominantly found in the brain environment and not in the extracranial environment. Therefore, it is surprising that *TMEM119* expression was observed to be decreased in intra- compared to extracranial metastases. Further experimental studies are required to investigate this observation.

In a next step, we analyzed the expression behavior of our candidate genes in relation to publicly available data of primary and metastatic melanoma from TCGA [42] to identify if the expression behavior of these genes was associated with patient survival. In total, 38 of our 103 candidate genes were measured in the public TCGA data set. We observed a significant association between gene expression and survival for 11 of those 38 genes (from most to least significant: *CXCL11*, *CCL8*, *ST6GALNAC5*, *PLA2G2D*, *KRT5*, *FGF7*, *CD38*, *CD48*, *CD3D*, *HSD11B1*, *CD8B*). All of these genes showed decreased expression in our intracranial melanoma metastases compared to their corresponding extracranial metastases. In the context of the TCGA cohort, reduced expression of ten of these genes was associated with significantly shorter survival compared to patients who showed increased expression. Thus, these genes may also have the potential to contribute to the poor prognosis of intracranial metastases. Most of these survival-associated genes are involved in the regulation of immune responses. This finding supports the generally known association between immune infiltration and survival for metastatic melanoma patients [67, 68]. However, there were also three other survival-associated genes that are involved in cell metabolism (*HSD11B1*, *ST6GALNAC5*) and cell growth (*FGF7*). We also found genes that are associated with melanoma metastases formation (*CCL8*, *CD38*) [55, 57] and melanoma cell migration and invasion (*KRT5*) [58]. Thus, the significant association between the expression of candidate genes and patient survival suggests that the expression behavior of these genes may also contribute to the poor prognosis of intracranial melanoma metastases. Still, a limitation of our survival analysis is that only 30 of the included TCGA patient samples were from distant metastases, whereas the majority of samples were from regional metastases. All metastases in our cohort were distant metastases, but a robust and potentially best matching survival analysis that would only consider the 30 distant metastases from TCGA was not possible, because only seven patients would have been in each group of the gene-specific survival comparison of the high and low expression group.

Further, several of the genes with increased expression in intra- compared to extracranial metastases predicted in at least 11 of 16 melanoma patients could potentially be of therapeutic relevance for the development of targeted treatment strategies for intracranial metastases (e.g. *PPBP*, *HEPACAM*, *SLC24A2*, *SLC38A11*, *FMN2*, *PMP2*). *PPBP*, also known as chemokine *CXCL7*, is involved in the stimulation of PI3K/Akt signaling [69] and known to promote breast cancer progression [70]. *CXCL7* can be targeted *in vitro*, *in vivo* and clinically by PI3K/Akt inhibitors [19, 71, 72]. Further, CSF1R inhibitors (GW2580) reduce myeloid cells in the tumor microenvironment of gliomas and significantly decrease the expression of chemokine *CXCL7*, thus inhibiting tumor growth [69, 73]. *HEPACAM* is involved in cell motility and cell-matrix interactions, known to control astrocyte self-organization and coupling [74], and able to suppress cancer cell growth and to induce migration [75]. Further analyses are necessary to analyze the role of the overexpression of *HEPACAM* in intracranial metastases to characterize its therapeutic potential. The two transporters *SLC24A2* and *SLC38A11* might be involved in metabolic reprogramming and adaptation to the brain-specific microenvironment [76]. Amino acid transporters are investigated in clinical trials and can be targets for cancer therapy [77, 78]. *FMN2* has essential roles in the organization of the actin cytoskeleton and cell polarity and has been reported to promote cell cycle arrest by inhibiting the degradation of the cyclin-dependent kinase inhibitor p21 [79]. Circular RNA of *FMN2* has been reported to play a role in colorectal cancer [80]. The role of the increased expression of *FMN2* in intracranial metastases should be analyzed by additional experiments. The myelin protein *PMP2* is involved in the regulation of melanoma cell invasion and may present a novel therapeutic target [51].

Finally, it is important to note that our study only includes a limited number of patients for which patient-matched metastases were available. This reduces the ability to generalize all findings, but the cohort size was still large enough to clearly demonstrate the need for a personalized analysis of the transcriptomes of the patient-matched metastasis pairs. Our predicted top candidate genes were in good accordance with three closely related melanoma metastasis studies and expression associations of candidate genes with patient survival indicate their clinical importance. Both things clearly support the relevance of our work and novel findings.

## Conclusions

Our computational analysis represents the first fully personalized analysis of transcriptomes of patient-matched melanoma metastasis pairs. Our findings contribute to a better characterization of genes and pathways that distinguish intra- from extracranial melanoma metastasis. Several of our findings are in good accordance with previously published melanoma metastasis studies. Especially the eight candidate genes that overlapped with the related studies and the eleven survival-associated candidate genes could provide an important basis for future experimental studies.

### Supplementary Information

**Additional file 1**: **Figure S1**: Dendrogram of the joint hierarchical clustering of individual melanoma metastases and normal tissue samples.

**Additional file 2**: **Figure S2**: Histogram of gene expression log_2_-ratios across all patient-matched intra- vs. extracranial melanoma metastasis pairs.

**Additional file 3**: **Figure S3**: Extended overview of signaling and immune pathway alterations for all patient-matched melanoma metastasis pairs.

**Additional file 4**: **Figure S4**: Comparison of the expression behavior of genes associated with the brain-like phenotype considering intracranial metastases and normal brain tissues.

**Additional file 5**: **Figure S5**: Expression behavior of the 103 candidate genes comparing intracranial metastases and normal brain tissues.

**Additional file 6**: **Table S1**: Gene expression data of all melanoma metastases.

**Additional file 7**: **Table S2**: Gene expression data of all normal tissues.

**Additional file 8**: **Table S3**: Overview of considered normal tissue samples.

**Additional file 9**: **Table S4**: Gene expression log_2_-ratio profiles of patient-matched intra- vs. extracranial melanoma metastasis pairs.

**Additional file 10**: **Table S5**: State-posterior decodings of the gene expression states by the HMM for all patient-matched melanoma metastasis pairs.

**Additional file 11**: **Table S6**: Signaling pathway gene annotations.

**Additional file 12**: **Table S7**: Immune pathway gene annotations.

**Additional file 13**: **Table S8**: Ranking of genes according to the number of melanoma metastasis patients with increased or decreased expression predictions by the HMM.

**Additional file 14**: **Table S9**: Gene ontology enrichment analysis results of genes with increased or decreased expression in intracranial metastases altered in at least 8 of 16 melanoma patients.

**Additional file 15**: **Table S10**: Survival data and gene expression group assignments of the TCGA melanoma patients for the 38 overlapping candidate genes.

**Additional file 16**: **Table S11**: Overlap of HMM gene expression state predictions of patient-matched and non-patient-matched melanoma metastasis pairs.

**Additional file 17**: **Table S12**: Overview comparing the expression behavior of the 103 top differentially expressed candidate genes to the top candidate genes of three related melanoma metastasis studies.

**Additional file 18**: **Table S13**: Additional meta-information regarding the considered melanoma metastasis patients.

**Additional file 19**: **Table S14**: Normalized TCGA gene expression levels of the 38 overlapping candidate genes considered for survival analysis.

**Additional file 20**: **Text S1**: Details to overrepresented signaling pathways for individual metastasis pairs.

**Additional file 21**: **Text S2**: Details to overrepresented immune pathways for individual metastasis pairs.

**Additional file 22**: **Text S3**: Details to RNA sequencing and preprocessing of reads.

## Data Availability

Gene expression profiles of all metastasis samples are provided in Additional file 6: Table S1. Gene expression profiles of all normal tissues are provided in Additional file 7: Table S2. Log_2_-ratio gene expression profiles of patient-matched melanoma metastasis pairs are provided in Additional file 9: Table S4. Signaling pathway annotations are provided in Additional file 11: Table S6 and immune pathway annotations are provided in Additional file 12: Table S7. Data underlying the TCGA-based survival analysis of our candidate genes are provided in Additional files 15 and 19: Tables S10 and S14. The R scripts used to perform the study and the Java JAR file of the utilized HMM implementation are freely available from GitHub under https://github.com/TheresaKraft/MelBrainSys_expression.

## Abbreviations

HMM: Hidden Markov Model
TCGA: The Cancer Genome Atlas
FDR: False discovery rate
