# Supplementary material for: Personalized identification and characterization of genome-wide gene expression differences between patient-matched intracranial and extracranial melanoma metastasis pairs
Source: Acta Neuropathol Commun. 2024 Apr 24;12:67. doi: 10.1186/s40478-024-01764-5 (PMC11055243; doi:10.1186/s40478-024-01764-5)
Supplement: Supplementary file 1 — Additional file 1: Figure S1: Dendrogram of the joint hierarchical clustering of individual melanoma metastases and normal tissue samples. [file 40478_2024_1764_MOESM1_ESM.pdf]

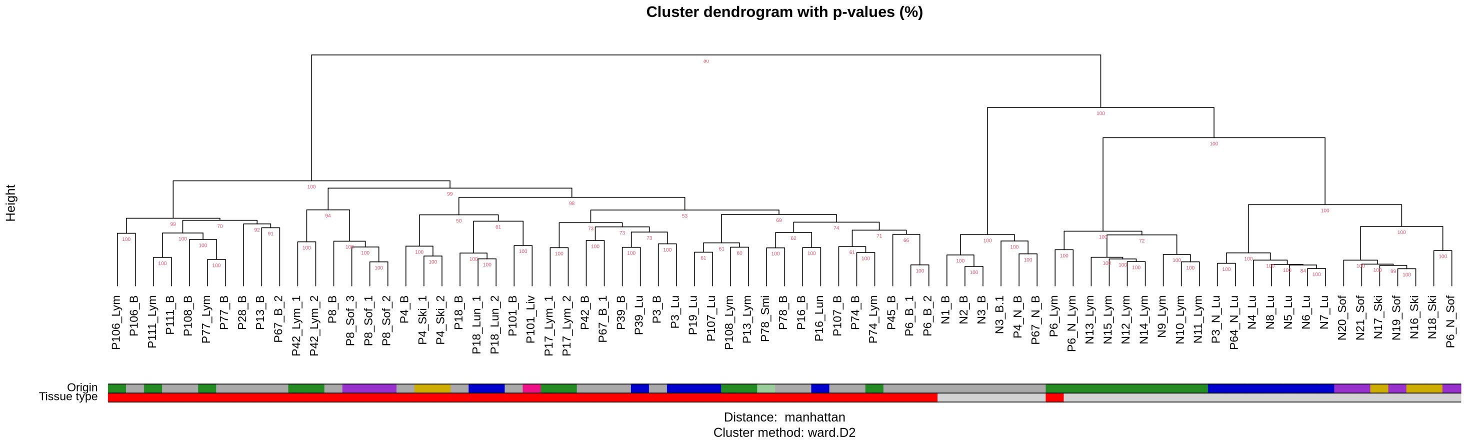

Figure S1: Joint hierarchical clustering of transcriptomes of all patient-matched melanoma metastases and normal tissue samples. The tissue type bar below the dendrogram represents the type of the sample either metastasis tissue (red, sample names starting with P) or normal tissue (grey, sample names starting with N). The origin of the tissue is represented by the origin bar below the dendrogram: brain (B, grey) lung (Lun, blue), lymph node (Lym, green), skin (Ski, yellow), liver (Liv, pink), small intestine (Smi, lightgreen) or soft tissue (Sof, purple). Stability of individual clusters is quantified by the red AU-value, where 100 means that a cluster was perfectly stable. A clear separation between metastasis and normal tissue samples is observed, except for the lymph node metastasis of patient P6.
