# Supplementary material for: Personalized identification and characterization of genome-wide gene expression differences between patient-matched intracranial and extracranial melanoma metastasis pairs
Source: Acta Neuropathol Commun. 2024 Apr 24;12:67. doi: 10.1186/s40478-024-01764-5 (PMC11055243; doi:10.1186/s40478-024-01764-5)
Supplement: Supplementary file 2 — Additional file 2: Figure S2: Histogram of gene expression log2-ratios across all patient-matched intra- vs. extracranial melanoma metastasis pairs. [file 40478_2024_1764_MOESM2_ESM.pdf]

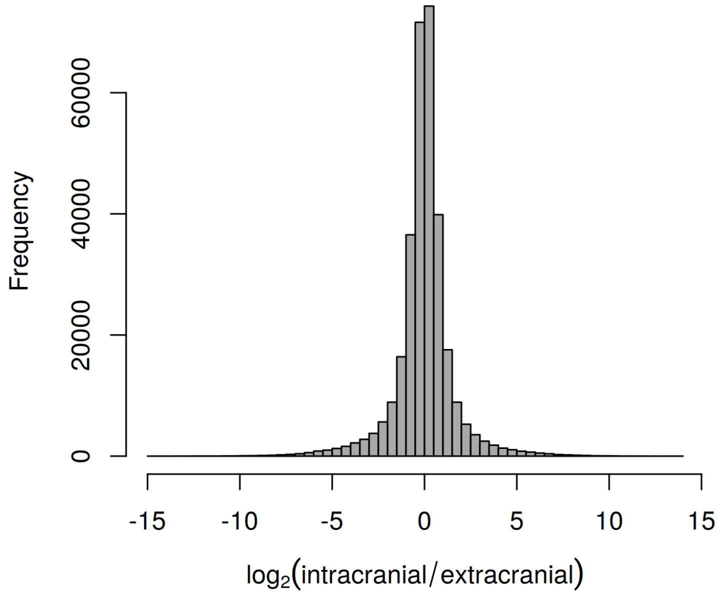

Figure S2: Histogram of gene expression $\log_2$-ratios across all patient-matched intra- vs. extracranial melanoma metastases. The majority of genes has unchanged expression levels with values about zero. Down- and up-regulated genes in intracranial metastases have expression levels clearly different from zero.
