# Supplementary material for: Personalized identification and characterization of genome-wide gene expression differences between patient-matched intracranial and extracranial melanoma metastasis pairs
Source: Acta Neuropathol Commun. 2024 Apr 24;12:67. doi: 10.1186/s40478-024-01764-5 (PMC11055243; doi:10.1186/s40478-024-01764-5)
Supplement: Supplementary file 3 — Additional file 3: Figure S3: Extended overview of signaling and immune pathway alterations for all patient-matched melanoma metastasis pairs. [file 40478_2024_1764_MOESM3_ESM.pdf]

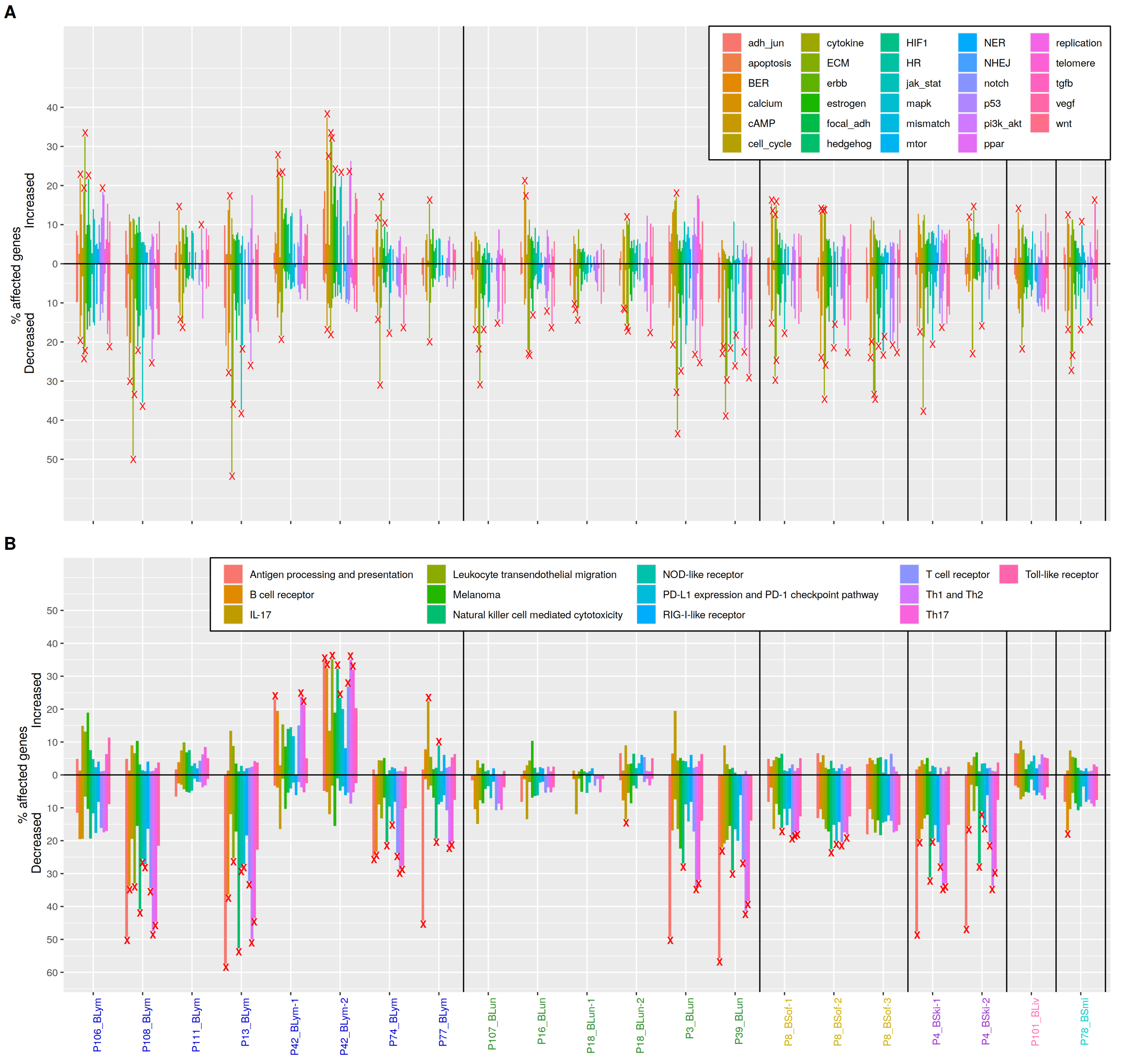

Figure S3: Signaling and immune pathway alterations for each patient-matched melanoma metastasis pair. A, Percentage of genes in each cancer-relevant signaling pathway that were either increased or decreased expressed in the intracranial metastasis compared to the corresponding extracranial metastasis of a patient-matched metastasis pair. B, Percentage of genes in each immune-relevant pathway that were either increased or decreased expressed in the intracranial metastasis compared to the corresponding extracranial metastasis of a patient-matched metastasis pair. Significant enrichment of decreased or increased expressed genes in pathways of subpanels A and B are marked with 'x' (FDR-adjusted p-value < 0.05).
