# Supplementary material for: Personalized identification and characterization of genome-wide gene expression differences between patient-matched intracranial and extracranial melanoma metastasis pairs
Source: Acta Neuropathol Commun. 2024 Apr 24;12:67. doi: 10.1186/s40478-024-01764-5 (PMC11055243; doi:10.1186/s40478-024-01764-5)
Supplement: Supplementary file 4 — Additional file 4: Figure S4: Comparison of the expression behavior of genes associated with the brain-like phenotype considering intracranial metastases and normal brain tissues. [file 40478_2024_1764_MOESM4_ESM.pdf]

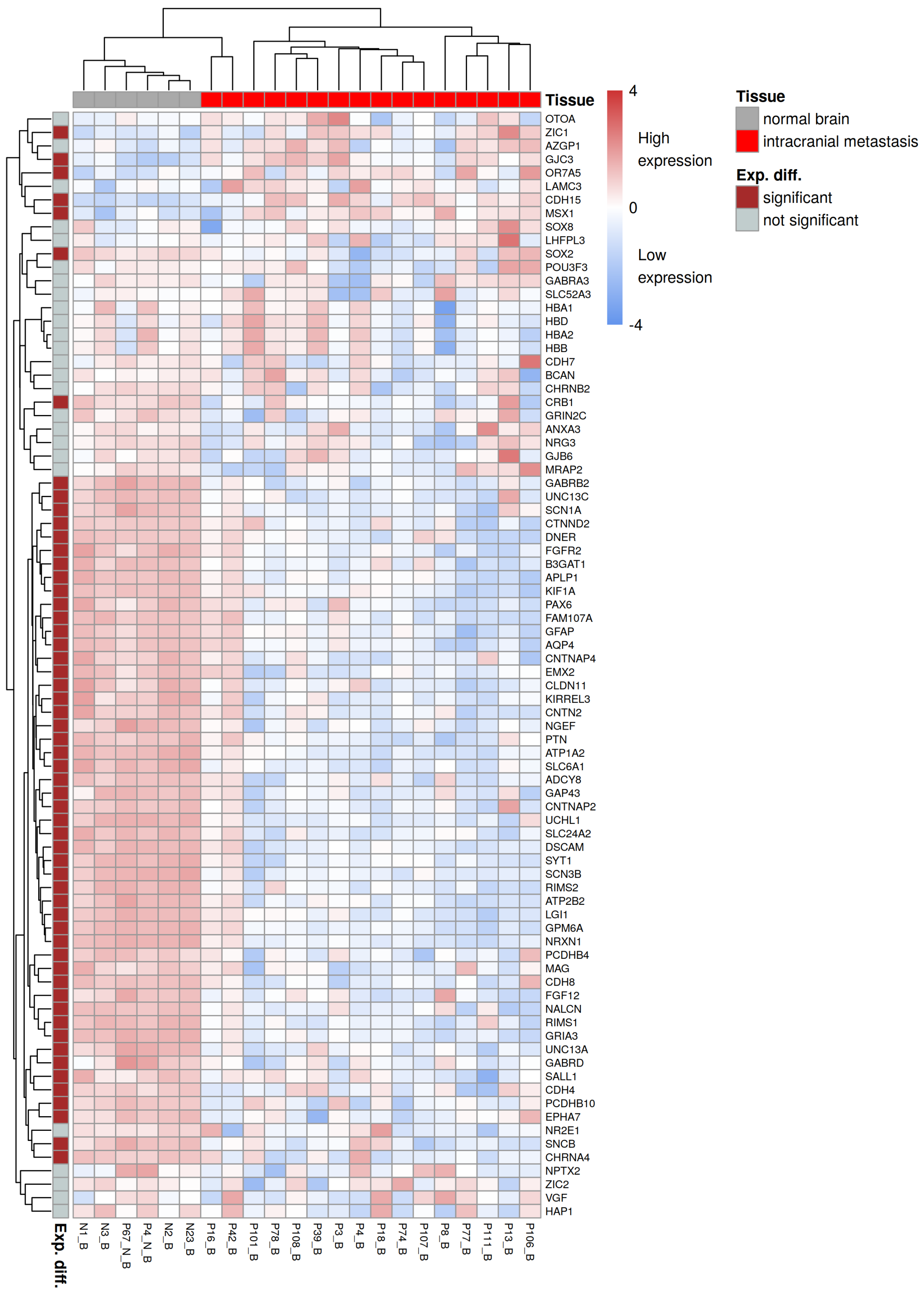

Figure S4: Heatmap of gene expression levels for intracranial metastases and normal brain samples based on genes that were associated with the brain-like phenotype. The columns of the heatmap represent the normal brain samples (tissue: dark grey) and the intracranial metastases (tissue: red). The rows of the heatmap represent the expression levels of the individual genes. A color gradient from blue over white to red is used to visualize the z-score scaled $\log_2$-gene expression levels of the individual genes. Significant expression differences of genes between the intracranial metastases and the normal brain tissues were determined by a standard differential gene expression analysis using the R package limma. The results are highlighted left to the heatmap (significant expression difference with FDR-adjusted p-value < 0.05: dark red). The hierarchical clustering of the rows and columns was done based on the Manhattan distance in combination with complete linkage. The genes associated with the brain-like phenotype that formed the basis of the heatmap were associated with the GO terms shown in Figure 5B of the main manuscript. The majority of genes (52 of 82) have significantly lower expression levels in the intracranial metastases compared to the normal brain tissues, but there are also some genes (5 of 82) with significantly greater expression levels in intracranial metastases and several genes with similar expression levels in intracranial metastases and normal brain tissues (25 of 82). This indicates that our observed brain-like phenotype is potentially jointly driven by normal cells in the metastases microenvironment and tumor cells of the intracranial metastases.
