# Supplementary material for: Personalized identification and characterization of genome-wide gene expression differences between patient-matched intracranial and extracranial melanoma metastasis pairs
Source: Acta Neuropathol Commun. 2024 Apr 24;12:67. doi: 10.1186/s40478-024-01764-5 (PMC11055243; doi:10.1186/s40478-024-01764-5)
Supplement: Supplementary file 5 — Additional file 5: Figure S5: Expression behavior of the 103 candidate genes comparing intracranial metastases and normal brain tissues. [file 40478_2024_1764_MOESM5_ESM.pdf]

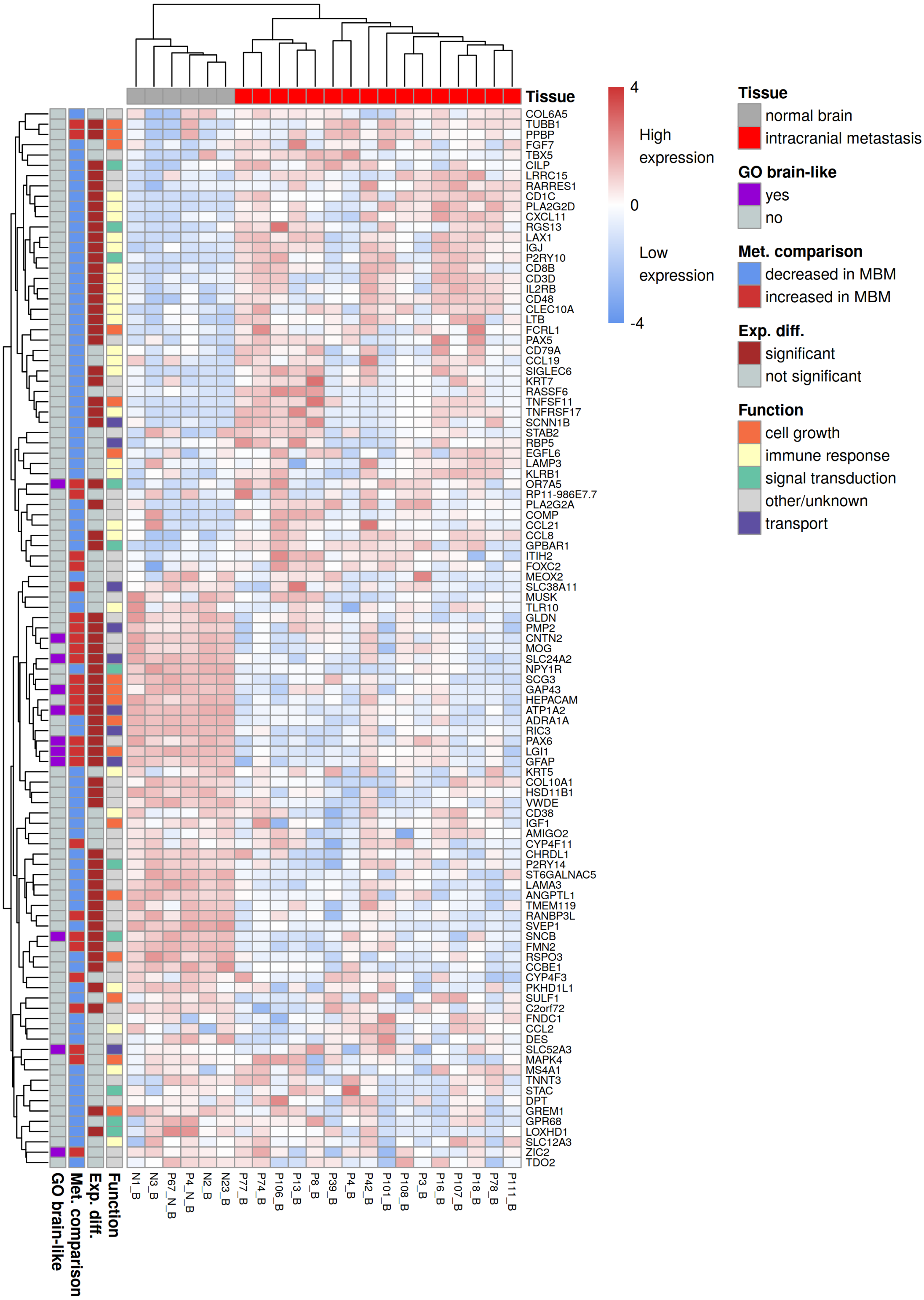

Figure S5: Heatmap of gene expression levels for intracranial metastases and normal brain samples based on genes that were predicted to be down- or upregulated in the patient-matched intra- and extracranial metastasis pairs of at least 11 patients from Figure 6 of the main manuscript. The columns of the heatmap represent the normal brain samples (tissue: dark grey) and the intracranial metastases (tissue: red). The rows of the heatmap show the expression levels of the individual genes. A color gradient from blue over white to red is used to visualize the z-score scaled $\log_2$-gene expression levels of the individual genes. Significant expression differences of genes between the intracranial metastases and the normal brain tissues are highlighted left to the heatmap based on a standard differential gene expression analysis using the R package limma (significant expression difference with FDR-adjusted p-value < 0.05: dark red in Exp. diff. annotation column). The left annotation block of the heatmap further shows the general function of individual genes, the corresponding expression state of each gene in the patient-matched intra- vs. extracranial metastasis pairs from Figure 6, and if a gene was part of the gene set that was associated with the observed brain-like phenotype. Many of the candidate genes show significant expression differences between the intracranial metastases and the normal brain tissues (Exp. diff. column: dark red), but there are also many genes that are expressed at the same level in intracranial metastases and normal brain tissues (Exp. diff. column: grey). This indicates how tumor cells of the intracranial metastases and normal cells of the microenvironment may express individual genes. The hierarchical clustering of the rows and columns was done based on the Manhattan distance in combination with complete linkage.
