# Supplementary material for: Personalized identification and characterization of genome-wide gene expression differences between patient-matched intracranial and extracranial melanoma metastasis pairs
Source: Acta Neuropathol Commun. 2024 Apr 24;12:67. doi: 10.1186/s40478-024-01764-5 (PMC11055243; doi:10.1186/s40478-024-01764-5)
Supplement: Supplementary file 20 — Additional file 20: Text S1: Details to overrepresented signaling pathways for individual metastasis pairs. [file 40478_2024_1764_MOESM20_ESM.pdf]

Personalized identification and characterization of genome-wide gene expression differences between patient-matched intracranial and extracranial melanoma metastasis pairs

Text S1: Characteristic expression alterations of cancer-relevant signaling pathways of individual patient-matched metastasis pairs

The following points summarize details to specific signaling pathway overrepresentations of individual metastasis pairs based on Figure 3B of the main manuscript.

- The cytokine-receptor interaction pathway was enriched for differentially expressed genes in all metastasis pairs (Figure 3B, cytokine: blue bars). In more detail, all metastasis pairs were enriched for decreased expression and seven pairs were additionally enriched for increased expression in the intra- compared to the extracranial metastasis (P106_BLym, P18_BLun-2, P3_BLun, P42_BLym-2, P77_BLym, P8_BSof-1, P8_BSof-2).

- The calcium signaling pathway was significantly enriched for differentially expressed genes in 19 of 21 pairs (Figure 3B, calcium: orange bars). Calcium signaling has recently been added to KEGG pathways in cancer and has been reported to play an important role in gliomas [Hausmann et al., 2023]. Enrichment of increased and decreased expression of calcium signaling pathway genes in intracranial metastases was observed for five metastasis pairs (P106_BLym, P42_BLym-2, P78_BSmi, P8_BSof-1, P8_BSof-2). Five other metastasis pairs only showed enrichment of increased expression in the intracranial metastases (P101_BLiv, P111_BLym, P16_BLun, P4_BSki-2, P42_BLym-1), and the intracranial metastases of eight metastasis pairs were enriched for decreased expression of calcium signaling genes (P107_BLun, P108_BLym, P13_BLym, P18_BLun-1, P18_BLun-2, P3_BLun, P39_BLun, P8_BSof-3). Only two metastasis pairs showed no enrichment of differential expression of calcium signaling pathway genes (P74_BLym and P77_BLym).

- The ECM-receptor interaction pathway was significantly enriched for differential expression in 17 metastasis pairs (Figure 3B, ECM: pink bars). Enrichment of increased and decreased expression of ECM signaling genes in the intra- compared to the extracranial metastasis was observed for two metastasis pairs (P106_BLym, P08_BSof-1). Four metastasis pairs showed enrichment of increased expression in the intracranial metastases (P4_BSki-2, P42_BLym-1, P42_BLym-2, P74_BLym) and a significant enrichment of decreased expression of ECM signaling genes was observed for 11 pairs (P107_BLun, P108_BLym, P13_BLym, P16_BLun, P18_BLun-1, P18_BLun-2, P3_BLun, P39_BLun, P78_BSmi, P8_BSof-2, P8_BSof-3).

- The cAMP signaling pathway was found to be enriched in 12 metastasis pairs (Figure 3B, cAMP: light green bars). The cAMP-dependent pathway is a second-messenger pathway which can regulate cancer cell growth, invasion and migration [Zhang et al., 2020]. One pair showed enrichment of differentially expressed cAMP pathway genes in both directions (P74_Blym). An enrichment of increased expression in the intra- compared to

the extracranial metastasis was found for six pairs (P13_BLym, P16_BLun, P42_BLym-1, P42_BLym-2, P8_BSof-1, P8_Bsof-2). An enrichment of cAMP signaling with decreased expression in the intra- compared to the extracranial metastasis was observed for five patient-matched metastasis pairs (P111_BLym, P18_BLun-1, P18_BLun-2, P39_BLun, P8_BSof-3).

- The PI3K/Akt signaling pathway showed a significant enrichment of differentially expressed genes in 12 metastasis pairs (Figure 3B, PI3K/Akt: petrol bars). An enrichment of PI3K/Akt signaling genes with increased expression in the intra- compared to the extracranial metastasis was observed for three patient-matched metastasis pairs (P106_BLym, P111_BLym, P42_Blym-2), and an enrichment of PI3K/Akt signaling genes with decreased expression was observed for nine pairs (P107_BLun, P108_BLym, P13_BLym, P16_BLun, P3_BLun, P39_BLun, P4_BSki-1, P78_BSmi, P8_BSof-3).

- The Jak-STAT signaling pathway was enriched for decreased expression in the intra- compared to the extracranial metastasis of ten patient-matched metastasis pairs (Figure 3B, Jak-STAT: turquoise bars, P108_BLym, P13_BLym, P39_BLun, P4_BSki-1, P4_BSki-2, P74_BLym, P78_BSmi, P8_BSof-1, P8_BSof-2, P8_BSof-3). Interestingly, Jak-STAT signaling genes were not significantly enriched for increased expression in the intracranial metastasis of any patient-matched metastasis pair. Thus, a downregulation of Jak-STAT signaling in intracranial metastases could at least for some patient-matched pairs be a potential molecular feature to manifest differences between intra- and extracranial metastases.

References

[Hausmann et al., 2023] Hausmann, D., Hoffmann, D. C., Venkataramani, V., Jung, E., Horschitz, S., Tetzlaff, S. K., et al. (2023). Autonomous rhythmic activity in glioma networks drives brain tumour growth. *Nature*, 613(7942):179–186.

[Zhang et al., 2020] Zhang, H., Kong, Q., Wang, J., Jiang, Y., and Hua, H. (2020). Complex roles of cAMP-PKA-CREB signaling in cancer. *Exp. Hematol. Oncol.*, 9(1):32.
