# Supplementary material for: Personalized identification and characterization of genome-wide gene expression differences between patient-matched intracranial and extracranial melanoma metastasis pairs
Source: Acta Neuropathol Commun. 2024 Apr 24;12:67. doi: 10.1186/s40478-024-01764-5 (PMC11055243; doi:10.1186/s40478-024-01764-5)
Supplement: Supplementary file 21 — Additional file 21: Text S2: Details to overrepresented immune pathways for individual metastasis pairs. [file 40478_2024_1764_MOESM21_ESM.pdf]

Personalized identification and characterization of genome-wide gene expression differences between patient-matched intracranial and extracranial melanoma metastasis pairs

Text S2: Characteristic expression alterations of immune-relevant pathways in individual patient-matched metastasis pairs

The following points summarize details to specific immune pathway overrepresentations of individual metastasis pairs based on Figure 3C of the main manuscript.

- Nature killer cell mediated cytotoxicity genes were enriched for differential expression in 12 metastasis pairs. An enrichment of increased expression was only observed for P42_BLym-2 and an enrichment of decreased expression was found for 11 other metastasis pairs (P106_BLym, P108_BLym, P13_BLym, P3_BLun, P39_BLun, P4_BSki-1, P4_BSki-2, P74_BLym, P77_BLym, P8_BSof-1, P8_BSof-2).
- Genes of the TH17 cell differentiation pathway were significantly enriched for differential expression in 12 patient-matched metastasis pairs. Both metastasis pairs of patient P42 (P42_BLym-1, P42_BLym-2) were enriched for increased expression of TH17 pathway genes. Enrichment of decreased expression of TH17 pathway genes was found for ten metastasis pairs (P108_BLym, P13_BLym, P3_BLun, P39_BLun, P4_BSki-1, P4_BSki-2, P74_BLym, P77_BLym, P8_BSof-1, P8_BSof-2).
- Further, genes of the Th1 and Th2 cell differentiation pathway were enriched for differential expression in 11 metastasis pairs. Again, both metastases of patient P42 (P42_BLym-1, P42_BLym-2) were enriched for increased expression of TH1 and TH2 pathway genes. Decreased expression of TH1 and Th2 pathway genes was enriched for nine metastasis pairs (P108_BLym, P13_BLym, P3_BLun, P39_BLun, P4_BSki-1, P4_BSki-2, P74_BLym, P77_BLym, P8_BSof-1).
- Finally, genes of the antigen processing and presentation pathways were enriched for differential expression in ten metastasis pairs. Again, patient P42 also showed an enrichment of genes with increased expression for both of its metastasis pairs (P42_BLym-1, P42_BLym-2). Additionally, enrichment for decreased expression was observed for eight metastasis pairs (P108_BLym, P13_BLym, P3_BLun, P39_BLun, P4_BSki-1, P4_BSki-2, P74_BLym, P77_BLym).
