# Supplementary material for: Personalized identification and characterization of genome-wide gene expression differences between patient-matched intracranial and extracranial melanoma metastasis pairs
Source: Acta Neuropathol Commun. 2024 Apr 24;12:67. doi: 10.1186/s40478-024-01764-5 (PMC11055243; doi:10.1186/s40478-024-01764-5)
Supplement: Supplementary file 22 — Additional file 22: Text S3: Details to RNA sequencing and preprocessing of reads. [file 40478_2024_1764_MOESM22_ESM.pdf]

Personalized identification and characterization of genome-wide gene expression differences between patient-matched intracranial and extracranial melanoma metastasis pairs

Text S3: RNA sequencing and pre-processing of RNA-seq data

RNA sequencing of the metastasis samples from [Westphal et al., 2023] (first cohort) and of the newly sequenced metastasis samples (second cohort) was performed as follows:

- **RNA sequencing:** A tumor area of about 100 mm^2 was macrodissected from the marked metastasis tissue in one to fifteen 10-μm thick tissue sections. RNA was isolated using the RNeasy FFPE Kit (first cohort) and the miRNeasy FFPE Kit (second cohort) (Qiagen, Hilden, Germany). Quality assessment (DV200) was carried out on the 5200 Fragment Analyzer System using the standard-sensitivity RNA Kit (first cohort) and the RNA Kit (15NT) (second cohort) (both from Agilent Technologies, Santa Clara, CA). If DV200 > 25%, enrichment was performed employing the TruSeq RNA Access Library Prep Kit (first cohort) and the TruSeq RNA Exome Library Prep Kit (second cohort) (Illumina, San Diego, US-CA). According to the manufacturer's instructions, RNA input was based on the extent of fragmentation as determined by DV200 and ranged from 20 ng to 100 ng. RNA sequencing was performed on a NextSeq 500 (first cohort) and on a NextSeq 550 (second cohort) to obtain paired-end reads of 75 bp.

Pre-processing of the reads was done like reported in [Westphal et al., 2023]:

- **RNA-seq preprocessing:** The obtained reads were trimmed for quality and sequence adapters using Trimmomatic [Bolger et al., 2014]. The reads were aligned against the phase II reference of the 1000 Genomes Project, including decoy sequences d5 (hs37d5), using STAR [Dobin et al., 2013] in a two-pass mapping mode. First, an index was generated using the hs37d5 genome sequence and comprehensive gene annotations from Gencode (GRCh37.p13). This index was used for the initial mapping of the preprocessed reads. Next, all detected splice junctions from all samples were merged and used as a guide for the second mapping step. The read counts of all annotated genes were extracted from the alignments using the featureCounts function of the R subread package [Liao et al., 2013]. A joint principal component analysis (PCA) of the metastasis samples from the first and the second cohort showed that the metastasis samples of both cohorts were intermixed very well. Therefore, no additional batch correction was done.

References

[Bolger et al., 2014] Bolger, A., Lohse, M., and Usadel, B. (2014). Trimmomatic: a flexible trimmer for Illumina sequence data. *Bioinformatics*, 30:2114–2120.

[Dobin et al., 2013] Dobin, A., Davis, C., Schlesinger, F., Drenkow, J., Zaleski, C., Jha, S., et al. (2013). STAR: ultrafast universal RNA-seq aligner. *Bioinformatics*, 29:15–21.

[Liao et al., 2013] Liao, Y., Smyth, G., and Shi, W. (2013). The Subread aligner: fast, accurate and scalable read mapping by seed-and-vote. *Nucleic Acids Res*, 41:e108.

[Westphal et al., 2023] Westphal, D., Meinhardt, M., Grützmann, K., Schöne, L., Steininger, J., Neuhaus, L., et al. (2023). Identification of Epigenetically Regulated Genes Distinguishing Intracranial from Extracranial Melanoma Metastases. *Journal of Investigative Dermatology*, 143:1233–1245.
